# Silver-promoted solid-phase guanidinylation enables the first synthesis of arginine glycosylated Samoamide A cyclopeptide analogue

**DOI:** 10.3389/fchem.2022.1040216

**Published:** 2023-01-04

**Authors:** Bingxin Liang, Rong Li, Linji Li, Ming Tang, Xiang Li, Chunli Su, Hongli Liao

**Affiliations:** ^1^ School of Pharmacy, Chengdu Medical College, Chengdu, China; ^2^ School of Pharmacy, Second Military Medical University, Shanghai, China; ^3^ School of Public Health, Chengdu Medical College, Chengdu, China

**Keywords:** cyclopeptide synthesis, arginine N-glycosylation, solid-phase glycosylation, Samoamide A, antitumor biological activity

## Abstract

Cyclization and glycosylation serve as effective approaches for enhancing the drug properties of peptides. Distinct from typical glycosylation, atypical arginine N-glycosylation has drawn increasing attention due to its fundamental role in various cellular procedures and signaling pathways. We previously developed a robust strategy for constructing arginine N-glycosylated peptides characterized by silver-promoted solid-phase guanidinylation. Modeled after cyclic octapeptide Samoamide A, an antitumor peptide composed of eight hydrophobic amino acids extracted from cyanobacteria, herein we first performed arginine scanning to determine an optimal position for replacement with arginine. Consequently, the first synthesis of arginine glycosylated Samoamide A cyclopeptide analogue was described combining solid-phase glycosylation with solution-phase cyclization. The resultant SA-HH-TT displayed enhanced water solubility compared with the non-glycosylated SA-HH-TT. Notably, our method provides a universal strategy for synthesizing arginine N-glycosylated cyclopeptides.

## Introduction

Polypeptide drugs, a research hotspot in recent years, have been marketed as approximately 80 therapeutics worldwide due to their various biological activities, strong receptor affinity, low toxicity and low accumulation (Du QS et al., 2015; Muttenthaler et al., 2021), such as the well-known cyclosporin (Fahr, 1993), GLP-1 receptor agonists (Nauck et al., 2021) exenatide, liraglutide, lixiniatide, and the recently approved cyclopeptide drug Pegcetacoplan (Hoy, 2021). However, problems exist, including their short half-life and suboptimal stability, such as their susceptibility to proteolytic degradation, and their sensitivity to acid, alkali, high temperature or organic solvents can deactivate them (Smart et al., 2014). These defects have become tremendous obstacles to the clinical application of peptide drugs, therefore, peptide modification has been utilized as a top priority in the fight against the above shortcomings.

The common modification methods include modification of both ends of peptides, such as amidation and methylation (Chatterjee et al., 2013; Mura et al., 2016; Alonso-Bastida and Encarnacion-Guevara, 2019); introduction of non-toxic, non-immunogenic and highly water-soluble PEG groups (Pasut and Veronese, 2012); and cyclization strategies (Craik, 2006; Zorzi et al., 2017). Cyclic peptides are often more rigid than linear peptides and thus have strong resistance to the digestive system; they also have a large specific surface area, which provides high affinity and selectivity for protein targets.

Glycosylation is also one of the common modification methods. The composition of glycosyl can affect its key pharmacological and pharmacokinetic properties in terms of water solubility, stability or biological activity (Johnson et al., 2014). Almost all natural glycosylation modifications can be classified as N- or O-glycosides, among which O-glycosides are mainly linked to the hydroxyl groups of serine and threonine. In addition, a small amount of O-glycosylation occurs on tyrosine residues (Iwashkiw et al., 2013). The main connection mode of N-glycosides is that N-acetyl glucosamine is connected with the side chain of asparagine. It was later discovered that the NleB family of type III secretory system effector proteins from enteropathogenic *Escherichia coli* (EPEC) can induce a novel posttranslational modification of N-glycosylated proteins, arginine-N-acetyl glucosamine (Arg-N-GlcNAc), which can act on host death receptors, such as TNFR and FAS, and their connectors, such as FADD, TRADD, and RIPK1, and inhibit the death receptor-induced apoptosis and necrosis of host cells by blocking the formation of the DISC (death inducing signaling complex) complex mediated by the death domain (Li et al., 2013; Pearson et al., 2013; Ding et al., 2019).

In the method of chemical synthesis of glycopeptides, O-linked glycopeptides are mainly constructed by glycosylated amino acids and then introduced into polypeptides. For N-linked glycopeptides, the main research is focused on the glycosylation of asparagine. In addition to using preformed glycosyl amino acids to synthesize glycopeptides, we can also use convergence strategies to directly glycosylate peptide chains to produce glycosylated peptides, called Lansbury’s aspartylation (Li and Zhu, 2016; Lin and Liu, 2020). Later, with the discovery that arginine can also be induced glycosylation, researchers also began to study the method of synthesizing arginine glycosylation. Pan et al. (2014) developed a robust strategy for constructing Arg N-glycosylated peptides, which was characterized by the use of chemical synthesis to obtain feasible S-alkyl-isothiourea glycosyl donors and then through silver-promoted solid-phase guanidine acylation to synthesize Arg N-GlcNAc peptides. This method is suitable for the efficient preparation of glycopeptides at single or multiple Arg-GlcNAc sites. Similarly, Xiang Li and his partners used this silver-promoted solid-phase synthesis method to successfully obtain a kind of anti-rhamnosyl arginine specific antibody, which was able to diagnose infections caused by pathogens, such as *P. aeruginosa* or *N. meningitidis* (Li et al., 2016). Not only that, this method was also applied to the fully synthetic TRADD death domain (residues 195-312) with Arg235 N-GlcNAcylation. Wu et al. (2020) first obtained two longish peptidyl fragments of the wild-type primary sequence by microwave-assisted efficient solid-phase synthesis (SPPS). The N-GlcNAcylated portion was constructed by total synthesis and bound to the resin of an unprotected ornithine residue to achieve guanidinylation directly on the resin *via* a silver-promoted method. Then, the two fragments were linked by hydrazide-based native chemical ligation (NCL) to obtain Arg-GlcNAc TRADD (195-312). In addition to the synthesis of arginine-N-glycosylated glycopeptides, this method can also be applied to the synthesis of other peptides, such as stictamide A. According to retrosynthetic analysis, researchers found that it was not easy to obtain by either the liquid or solid-phase methods because of the N-prenyl-modified arginine. Therefore, inspired by the arginine-N-glycosylated glycopeptide synthesis method, they finally chose to directly synthesize the amino acid side chains on the solid-phase carrier, and the silver-promoted solid-phase synthesis method successfully synthesized stictamide A (Li et al., 2015). However, the application of silver-promoted solid-phase guanidinylation in cyclic peptides has not yet been reported.

**FIGURE 1:**
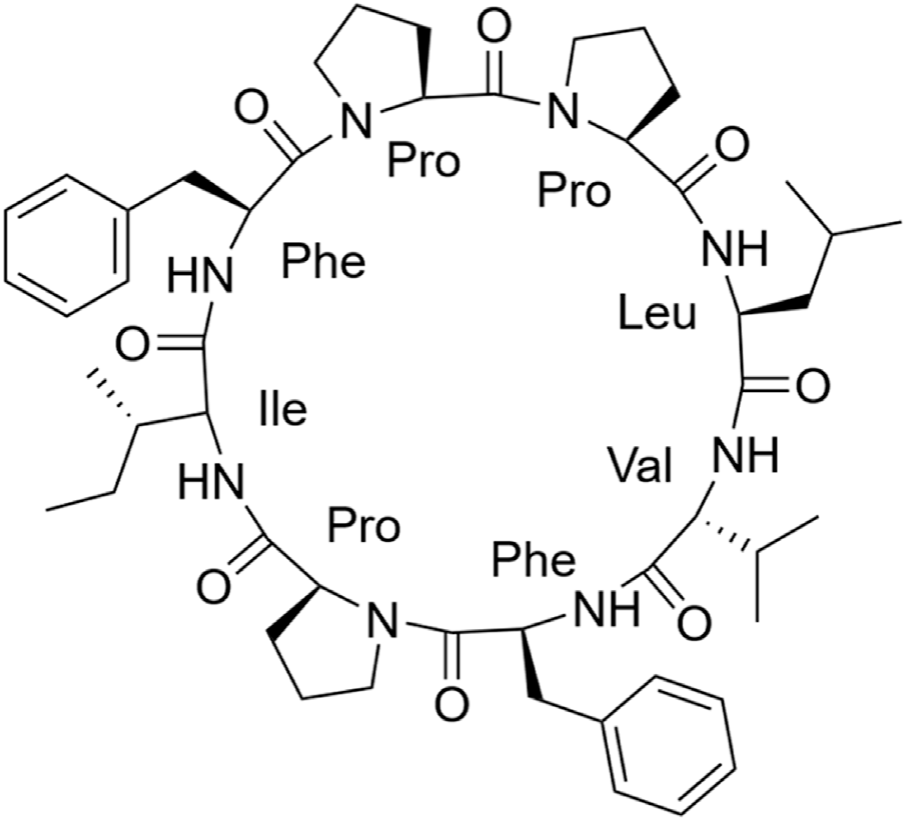
Structure diagram of Samoamide A.

**FIGURE 2:**
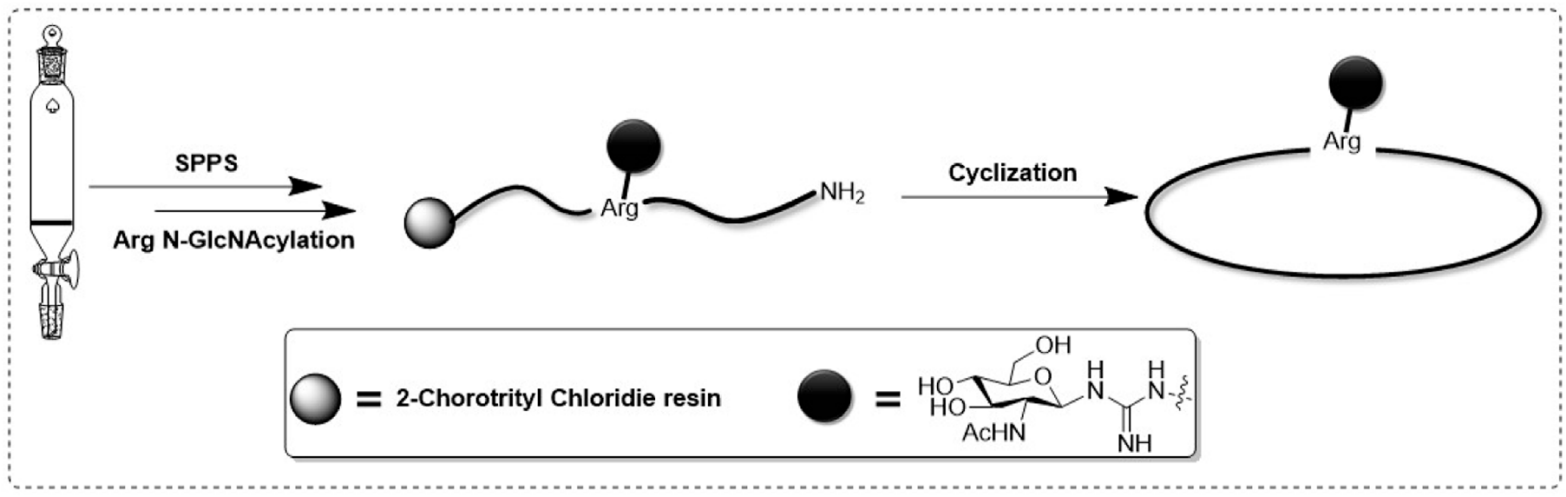
Synthesis of Arg-glycosylated cyclic peptides.

Samoamide A Figure 1 is an antitumor peptide consisting of eight hydrophobic amino acids extracted from cyanobacteria (Naman et al., 2017). Herein, using it as a template, after determining its inactive site by arginine scanning, the silver-promoted solid-phase arginine glycosylation method was applied to the cyclic peptide for the first time (Figure 2), and we designed and synthesized a series of Samoamide A derivatives and measured their activity to identify an excellent drug candidate for a novel antitumor drug.

## Experimental section

### General information

2-Chlorotrityl chloride resin (0.53 mmol·g^−1^ loading) was purchased from Jiangsu Shenlang Biotech Co., (Jiangsu, China). Fmoc-protected amino acids were purchased from Shanghai GL Biochem Ltd., and Jiangsu Shenlang Biotech Co., (Shanghai and Jiangsu, China). Phenol, DIC, TIPS, DIEA, HoBt, PyBOP, and HCTU were obtained from Adamas-beta (Shanghai, China). DMF, DCM, petroleum ether, ethyl ether, AgNO_3_, TFA, piperidine and other common reagents were purchased from CHRON Chemical Co., Ltd., (Chengdu, China). HCT116 cells were obtained from Shanghai Cellular Institute of Chinese Academy of Sciences (Shanghai, China). The CCK-8 value-added test kit was purchased from Shanghai Tongren Group (Shanghai, China). RPMI-1640, phosphate buffer solution, 0.25% trypsin, penicillin–streptomycin solution, and fetal bovine serum were purchased from Gibco Thermo Fisher Scientific (United States).

### Peptide synthesis

#### Synthesis of Samoamide A (SA-HH-0)

First, 2-chlorotrityl chloride resin (0.75 g, 0.53 mmol·g^−1^ loading capacity) was swollen with DCM (10 ml) for 25 min, mixed with Fmoc-Val-OH (2 mmol), DIEA (6 mmol) and DCM (8 ml), and added to the resin for 2 h to complete the coupling of the first amino acid, followed by washing with DMF (5 times), DCM (5 times) and DMF (2 times) to afford 1a. Then, the resin was treated with methanol for 20 min to block the residual active sites of the resin. Afterward, we deprotected Fmoc with 20% piperidine in DMF twice for 10 min every time to afford 1b. The steps of coupling, deprotection, and washing were repeated until all the amino acids of the Samoamide A sequence we designed were coupled to the resin to afford 1c. Reagents (88% TFA, 5% H_2_O, 5% phenol and 2% TIPS) were reacted at room temperature for 4 h to disconnect the crude straight-chain peptide from the resin. After filtering, a large amount of ethyl ether was added to crystallize and then centrifuged to obtain crude linear peptide 1d. PyBOP (3 eq), HoBt (3 eq) and DIPEA (6 eq) were dissolved in DCM and slowly added to the DCM solution of the peptide at 0 °C and stirred at room temperature for 14 h to afford crude oily Samoamide A 1e. Representative HPLC, HR-MS and NMR data are shown, and complete data can be found in the Supplementary Materials.

Cyclic peptides were purified by preparative high-performance liquid chromatography with a reverse C18 column and gradient elution (mobile phase A was 0.1% TFA in acetonitrile; mobile phase B was 0.1% TFA in water). SA-HH-0 [Cyclo-(VFPIFPPL)]. 58 mg, 15% purified yield. ESI-MS m/z calcd. For C_50_H_70_N_8_O_8_ 910.53; found [M + H]^+^ = 911.53; [M + Na]^+^ = 933.51.

#### Synthesis of arginine-scanned Samoamide A derivatives

The synthesis method of straight-chain peptide was the same as that of SA-HH-0, but the cleavage reagent was different. After obtaining Compound 2a, reagent (20% TFE, 80% DCM) was used to cleave the linear peptide. After concentration, a large amount of petroleum ether was added to crystallize, and then the liquid was removed by rotary steaming to obtain crude linear peptide 2b. PyBOP (3 eq), HoBt (3 eq) and DIPEA (6 eq) were dissolved in DCM, and the DCM solution of the peptide was slowly added to the DCM solution of PyBOP/HoBt/DIPEA at 0 °C and stirred at room temperature for 14 h to afford 2c. Then, the side chain protective group was removed, the reagents TFA and DCM were added, the volume ratio was 1:3, the reaction time was 4 h at room temperature, and we obtained the crude oily derivative 2d. The purification method was the same as that for SA-HH-0. Representative HPLC, HR-MS and NMR data are shown, and complete data can be found in the Supplementary Materials.

SA-HH-1 [Cyclo-(RFPIFPPL)]. 12 mg, 6% purified yield. ESI-MS m/z calcd. For C_51_H_73_N_11_O_8_ 967.56; found [M + H]^+^ = 968.56. SA-HH-2 [Cyclo-(VRPIFPPL)]. 8 mg, 4% purified yield. ESI-MS m/z calcd. For C_47_H_73_N_11_O_8_ 919.56; found [M + H]^+^ = 920.56. SA-HH-3 [Cyclo-(VFRIFPPL)]. 10 mg, 5% purified yield. ESI-MS m/z calcd. For C_51_H_75_N_11_O_8_ 969.568; found [M + H]^+^ = 970.57. SA-HH-4 [Cyclo-(VFPRFPPL)]. 12 mg, 6% purified yield. ESI-MS m/z calcd. For C_50_H_71_N_11_O_8_ 953.53; found [M + H]^+^ = 954.55. SA-HH-5 [Cyclo-(VFPIRPPL)]. 15 mg, 8% purified yield. ESI-MS m/z calcd. For C_47_H_73_N_11_O_8_ 919.56; found [M + H]^+^ = 920.49. SA-HH-6 [Cyclo-(VFPIFRPL)]. 20 mg, 10% purified yield. ESI-MS m/z calcd. For C_51_H_75_N_11_O_8_ 969.58; found [M + H]^+^ = 970.58. SA-HH-7 [Cyclo-(VFPIFPRL)]. 23 mg, 10% purified yield. ESI-MS m/z calcd. For C_51_H_75_N_11_O_8_ 969.58; found [M + H]^+^ = 970.58. SA-HH-8 [Cyclo-(VFPIFPPR)].18 mg, 9% purified yield. ESI-MS m/z calcd. For C_50_H_71_N_11_O_8_ 953.53; found [M + H]^+^ = 954.55.

#### Synthesis of arginine-glycosylated Samoamide A derivative (SA-HH-TT)

We also synthesized the linear peptide of SA-HH-TT according to the method of solid-phase synthesis to afford 3a and then used a reagent (10 ml DMF containing 2% NH_2_NH_2_) to remove the protective base Dde for 6 min in a constant temperature shaker at 30°C; this process was repeated 3 times. The samples were washed with DMF 3 times to afford 3b. After dissolving the glycosyl donor we prepared previously (0.6 mmol) and AgNO_3_ (0.6 mmol) with an appropriate amount of anhydrous DMF, Et_3_N (2 mmol) was added and reacted at room temperature for more than 12 h. After the reaction, the mixture was washed with DMF 5 times, MeOH 5 times and DCM 2 times. Linear glycopeptide 3c was cleaved with reagent (88% TFA, 5% H_2_O, 5% phenol and 2% TIPS) in the same way as arginine-scanned Samoamide A derivatives. After filtration, a large amount of ice diethyl ether was added for crystallization, centrifuged and blown with nitrogen to obtain crude linear peptide 3d. Then, using the same method as for 1e, cyclization yielded 3e. It was necessary to further remove the acetyl protective group from the glycosyl donor with methanol sodium methanol solution, react it at room temperature for 1 h, adjust the pH to neutral with dilute hydrochloric acid, and concentrate it to obtain glycosylated crude Samoamide A 3f. The purification method was the same as that for the other derivatives. Representative HPLC, HR-MS and NMR data are shown, and complete data can be found in the Supplementary Materials.

SA-HH-TT [Cyclo-(VFPIFPR(GlcNAc)L)]. 46 mg, 20% purified yield. ESI-MS m/z calcd. For C_59_H_88_N_12_O_13_ 1,172.66; found [M + H]^+^= 1,173.67; [M + Na]^+^= 1,195.65.

### Cell culture and cytotoxicity Assay

The human colon cancer cell line HCT116 was maintained in RPMI-1640 medium supplemented with 10% heat-inactivated FBS and 1% penicillin‒streptomycin at 37°C, 5% CO_2_, and complete humidification conditions. Cells (5 × 10^3^ cells/well) were inoculated in 96-well plates and treated with different concentrations of polypeptides for 48 h. The absorbance at 450 nm was then measured by adding the CCK8 kit, and the percentage of cell survival was calculated based on the ratio of the sample well to the reference well OD_450_ (Adan et al., 2016).

### Solubility test

Samoamide A was precisely weighed, and 1 mg was placed in a 10 ml centrifuge tube and diluted to scale with 95% ethanol. Then, 0, 200, 400, 600, 800, 1,000 μl aliquots were pipetted in a 5 ml centrifuge tube and adjusted to 1 ml with 95% ethanol, and the aqueous solutions were measured on a scale, sonicated for 5 min, and placed at room temperature. An additional 1 ml of 95% ethanol was added, and water was added to the same method as the blank. The absorbance values were measured at 214 nm to obtain the linear regression equation of the standard curve.

Sufficient amounts of Samoamide A and its glycosylated derivatives were dissolved in aqueous solutions at a constant temperature of 25°C, sonicated for approximately 10 min, centrifuged and allowed to settle to remove the insoluble matter from the solution, and the upper layer of supernatant was taken, that is, the saturated solution. After the saturated solution was properly diluted, its absorbance value was measured at 214 nm, and the concentration of the saturated solution was calculated by substituting the above equation (Chevalier et al., 2001).

## Results and discussion

### Synthesis of Samoamide A

**FIGURE 3:**
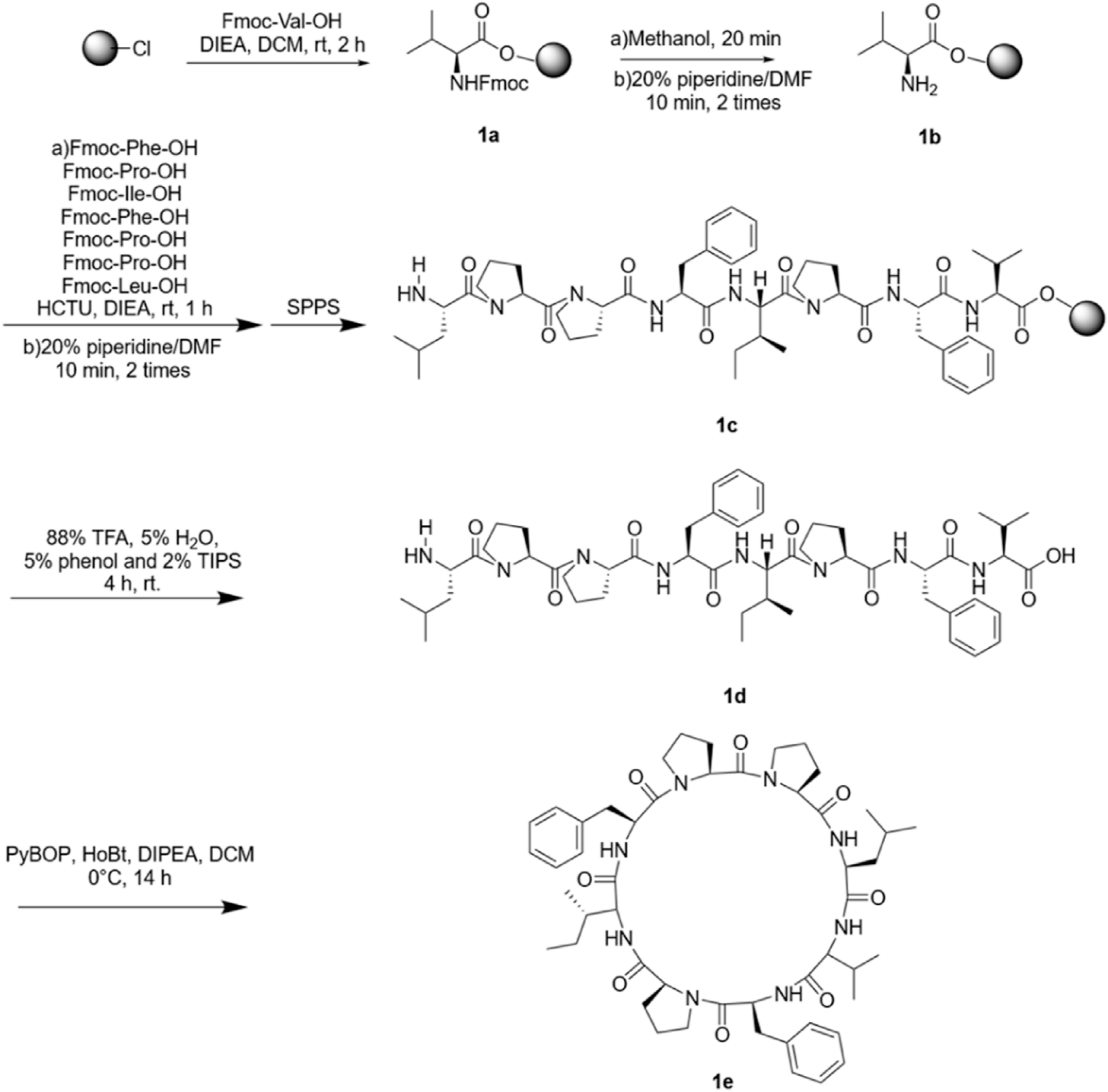
Synthesis route of SA-HH-0.

Samoamide A is cytotoxic to a variety of cancer cells, including non-small cell lung cancer cells and colorectal cancer cells (Naman et al., 2017; Chang et al., 2019). However, its low content and poor water solubility in nature limit its application in the development of new drugs. Hence, we manually synthesized Samoamide A by solid-phase synthesis (Figure 3). Using 2-chlorotrityl chloride resin as the carrier and HCTU and DIEA as the condensation system, the straight-chain peptide was synthesized by solid-phase synthesis. Then, PyBOP, HoBt, and DIPEA were used as cyclization systems and cyclized in DCM solution. Finally, we obtained Samoamide A, and its relative molecular weight was determined by mass spectrometry (Table 1).

**TABLE 1:** Detailed data of Samoamide A derivatives.

Compound	Sequence	HRMS,m/z [M + H]^+^	M	Retention time (min)
SA-HH-0	VFPIFPPL	911.53	910.53	28.41
SA-HH-1	RFPIFPPL	968.56	967.56	23.41
SA-HH-2	VRPIFPPL	920.56	919.56	24.05
SA-HH-3	VFRIFPPL	970.57	969.58	25.07
SA-HH-4	VFPRFPPL	954.55	953.53	22.50
SA-HH-5	VFPIRPPL	920.49	919.56	22.90
SA-HH-6	VFPIFRPL	970.58	969.58	24.95
SA-HH-7	VFPIFPRL	970.58	969.58	22.62
SA-HH-8	VFPIFPPR	954.55	953.53	23.52
SA-HH-TT	VITIFPR(GIcNAc)L	1,173.67	1,172.66	20.70

### Design and synthesis of arginine-scanned Samoamide A derivatives

**FIGURE 4:**
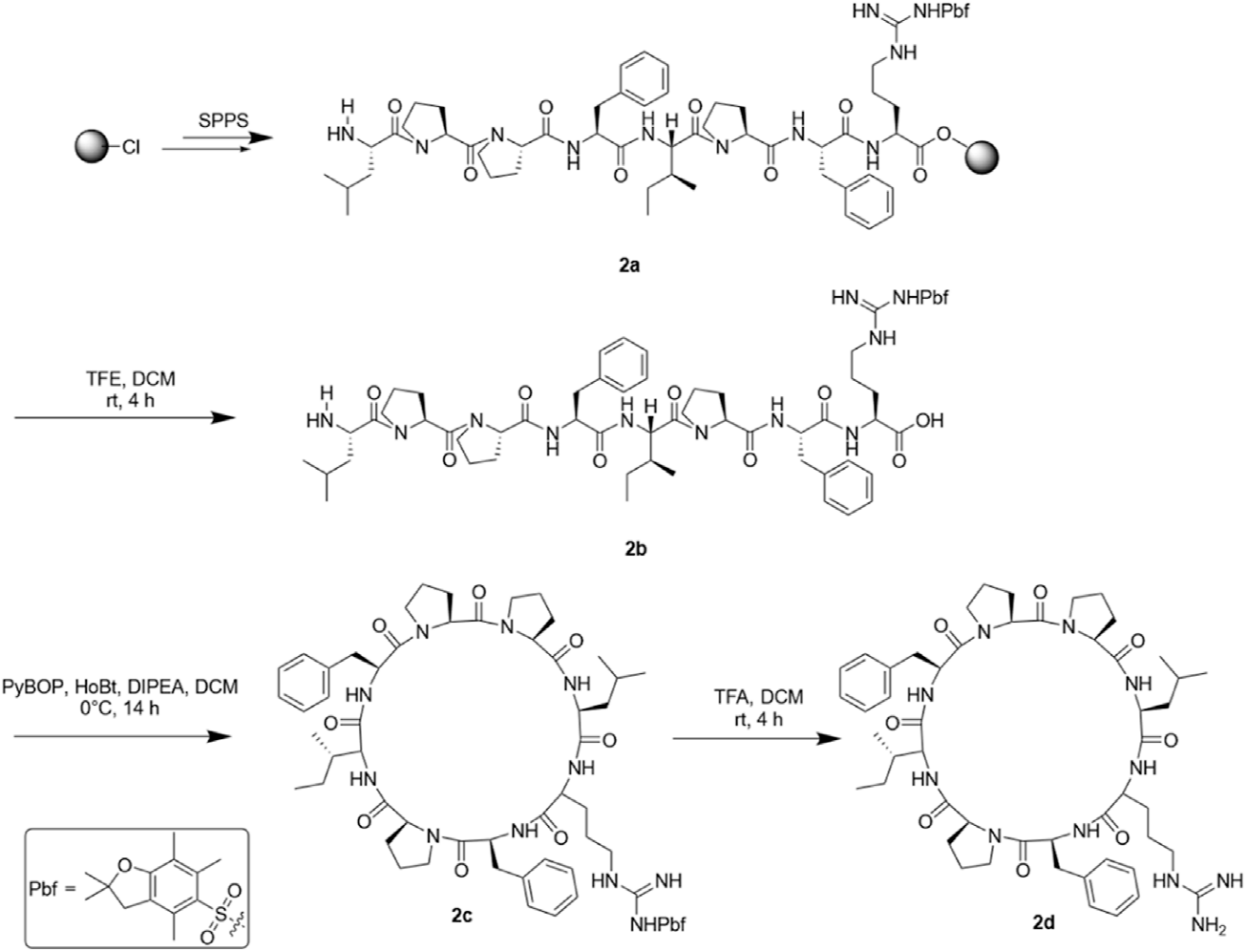
Synthesis route of SA-HH-1.

**FIGURE 5:**
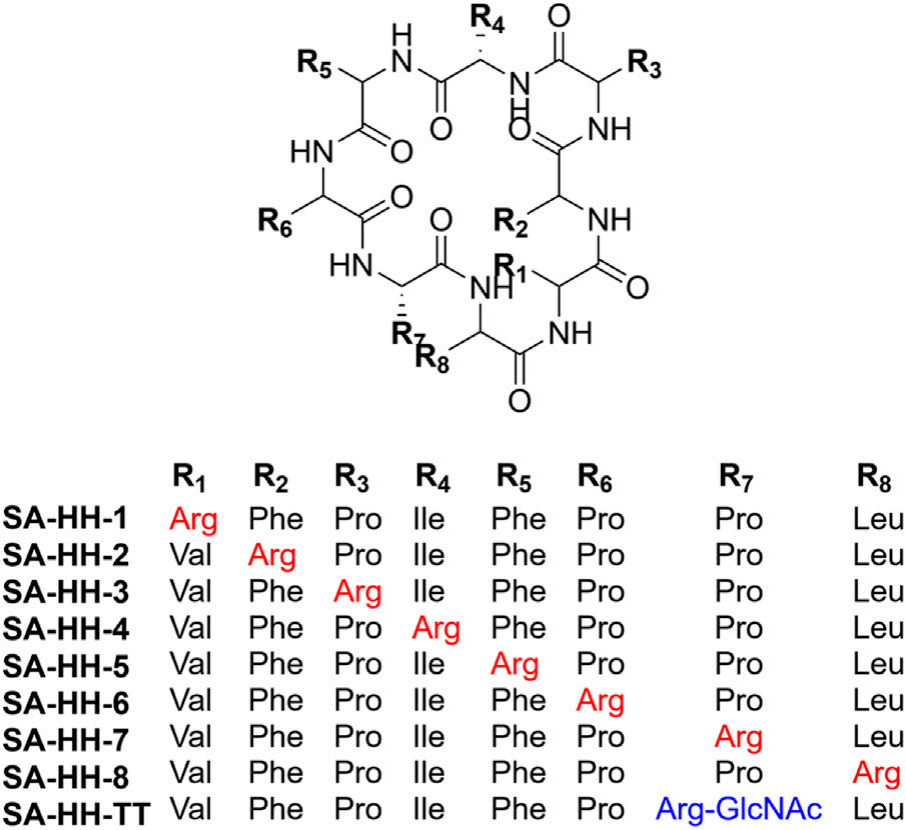
Structure diagram of Samoamide A derivatives.

Then, we scanned the cyclic octapeptide using arginine because the introduction of cationic amino acids can improve the properties of the peptide, but more importantly, to identify its inactive site and introduce arginine to guarantee further arginine-N-glycosylation. Taking the synthesis of SA-HH-1 as an example, the synthesis route is shown in Figure 4. Using 2-chlorotrityl chloride resin as the carrier and HCTU and DIEA as the condensation system, the arginine-scanned straight-chain peptide was synthesized by solid-phase synthesis. Then, PyBOP, HoBt, and DIPEA were used as cyclization systems and cyclized in DCM solution. Finally, we successfully obtained eight derivatives (Figure 5), and their relative molecular weights were determined by mass spectrometry (Table 1). Since Arg has two amino groups, when cyclizing, it should be noted that we need to retain the protective group of the side chain amino group and then remove the amino protection group of the arginine side chain group after cyclization so that the amino group at the end of the peptide chain is correctly connected to the carboxyl group; therefore, we chose TFE (Muhajir et al., 2021).

### Design and synthesis of an arginine-glycosylated Samoamide A derivative (SA-HH-TT)

**FIGURE 6:**
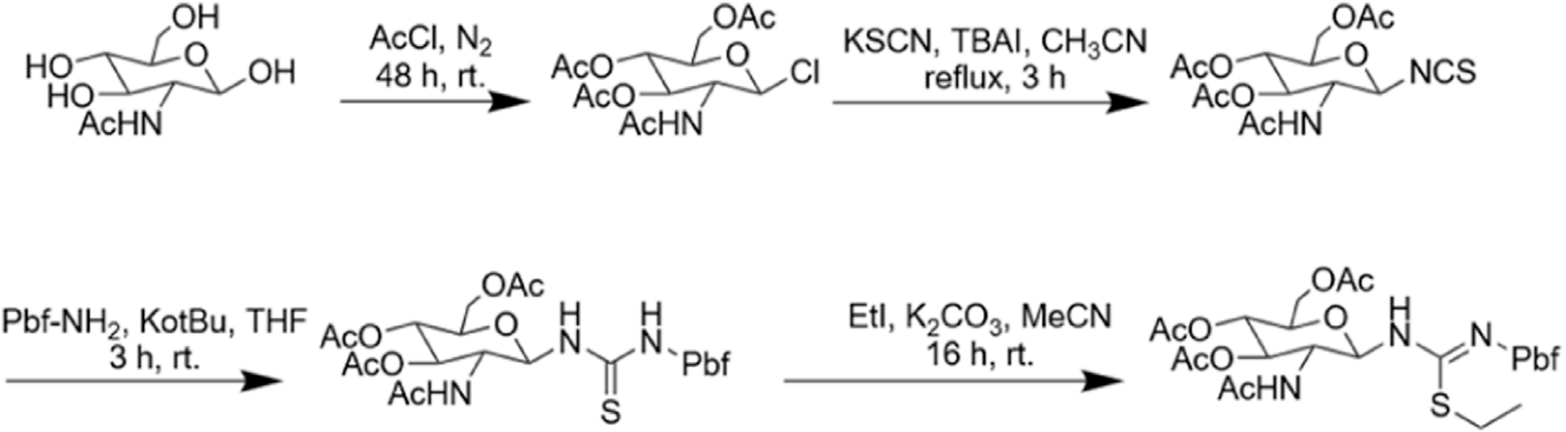
Synthesis routes of glycosyl donors.

**FIGURE 7:**
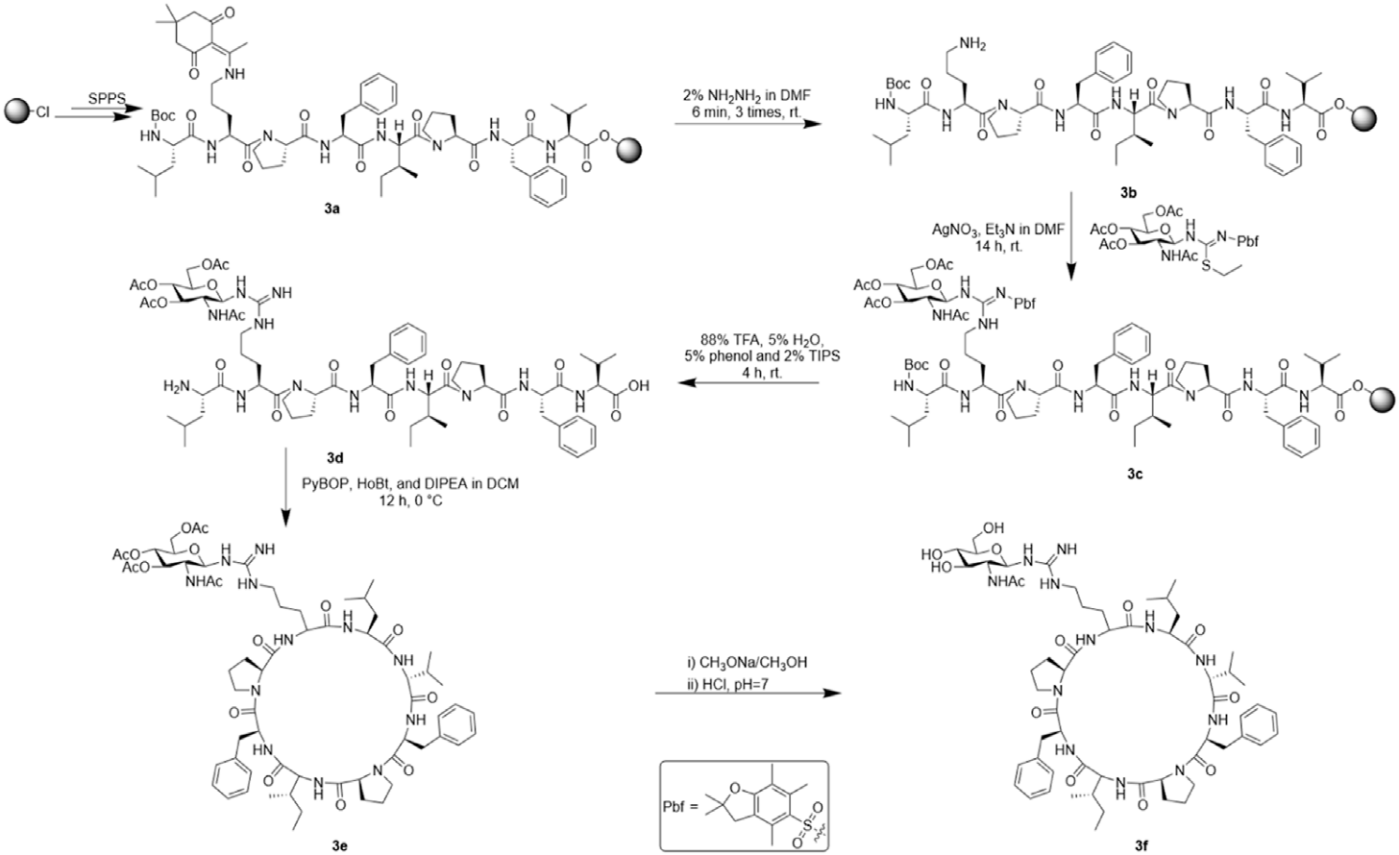
Synthesis route of SA-HH-TT.

When we identified the inactive site, we decided to further modify the arginine glycosylation at this site (Figure 5). Using the SSG method, we attached the preprepared S-alkyl isothiocyanate glycosyl donor (Figure 6) to ornithine, assembled the two parts, and achieved arginine glycosylation modifications (Figure 7). We confirmed the target product by mass spectrometry. In the process of obtaining the product, we found that in accordance with the Fmoc solid-phase synthesis method, when removing the ornithine side chain protection group, the use of NH_2_NH_2_ will also shed the Fmoc protection group of the amino acid at the end of the peptide chain so that the glycosyl donor cannot be correctly connected to the corresponding position. Using NH_2_OH HCl and imidazole (Díaz-Mochón et al., 2004), we were able to remove only the Dde protective group and retain the Fmoc protective group. After glycosylation, cyclization was carried out after the Fmoc protective base was removed. However, this method is cumbersome; therefore, we found another way to replace the last amino acid of the coupling with the amino acid protected by Boc because the Boc protective group is sensitive to acidity but is not sensitive to the alkaline environment. Therefore, when using NH_2_NH_2_, the Boc protective group will not fall off. When cutting the resin after glycosylation, the TFA can remove the Boc, and then the glycosylated straight chain is successfully cyclized. Similarly, to ensure the correct cyclization of cyclic peptides, we removed the hydroxyl protective acetyl group from the glycosyl donor after cyclization.

At present, the method of arginine glycosylation is roughly divided into two types: one is to construct glycosyl arginine and then introduce it into the solid-phase synthesis; the other is the direct solid-phase glycosylation of the peptide side chain functional group after the synthesis of the peptide chain (Wang et al., 2017; Rodriguez-Mayor et al., 2020). The limitation of the former is that after the construction of glycosyl arginine, a tedious purification step is needed, which can lead to a significant reduction in yield; the reaction also introduces Hg^2+^ harmful to the human body, which may limit the application of this method to arginine glycosylation (Rodriguez-Mayor et al., 2020). In the latter, in addition to the method described herein, silver-promoted direct solid-phase glycosylation is performed to obtain arginine glycosylated peptides (Pan et al., 2014); Yang et al. (2021) used palladium complexes as catalysts to achieve stereoselective arginine glycosylation. Different from our method, we combined thiourea glycosides and ornithine-bearing peptides, and they constructed glycopeptides directly from natural arginine peptides. However, to obtain high-yield products, a variety of complex metal palladium catalysts are needed, in which Pd(PPh_3_)_4_ is used, which is difficult to preserve. Some palladium catalysts are expensive and may even cause harm to the environment and the human body, limiting the development of their applications.

### Antitumor activity

**FIGURE 8:**
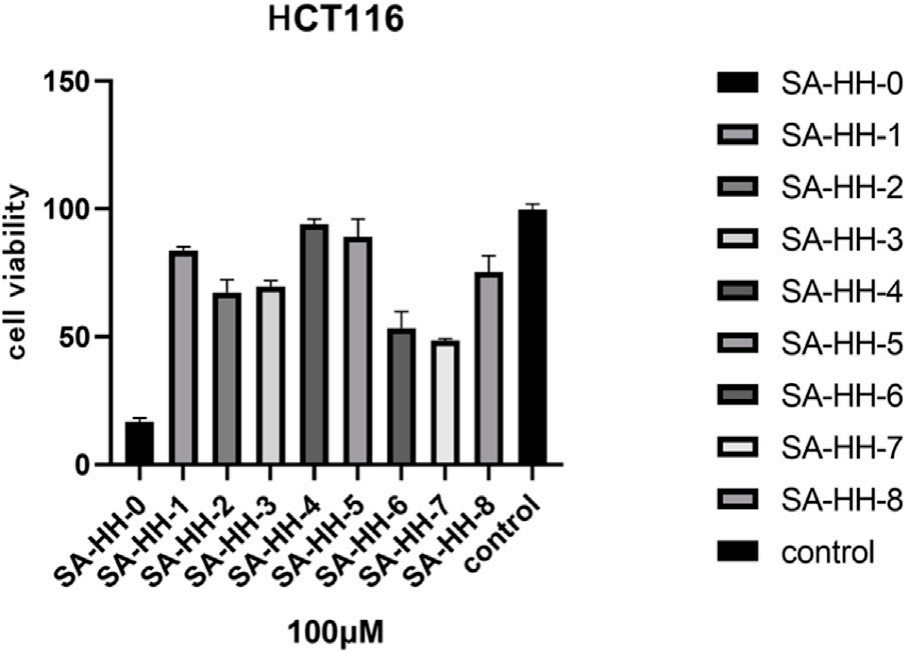
Effects of 100 μM Samoamide A derivatives on cell viability.

**FIGURE 9:**
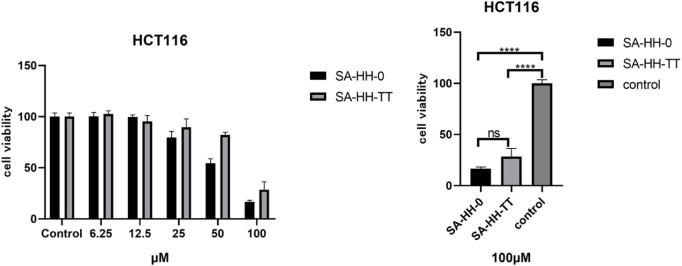
Effects of Samoamide A derivatives on cell viability.

The activity of the derivatives scanned by arginine was determined, and the inactive site of cyclic octapeptide was confirmed again (Figure 8). However, in the cytotoxicity test of arginine-glycosylated Samoamide A, we regrettably found that we did not obtain new compounds with better activity or the same activity as we expected (Figure 9), compared to the parent peptide, the activity decreased slightly. At 100 μM, the survival rate of cells treated with Samoamide A was 17%, while that treated with SA-HH-TT was 28%. We speculate that steric hindrance may fold the structure differently from the original structure, affecting its antitumor activity. However, the specific cause requires further confirmation. Representative activity data are shown, and complete data can be found in the Supplementary Materials.

### Solubility test

**FIGURE 10:**
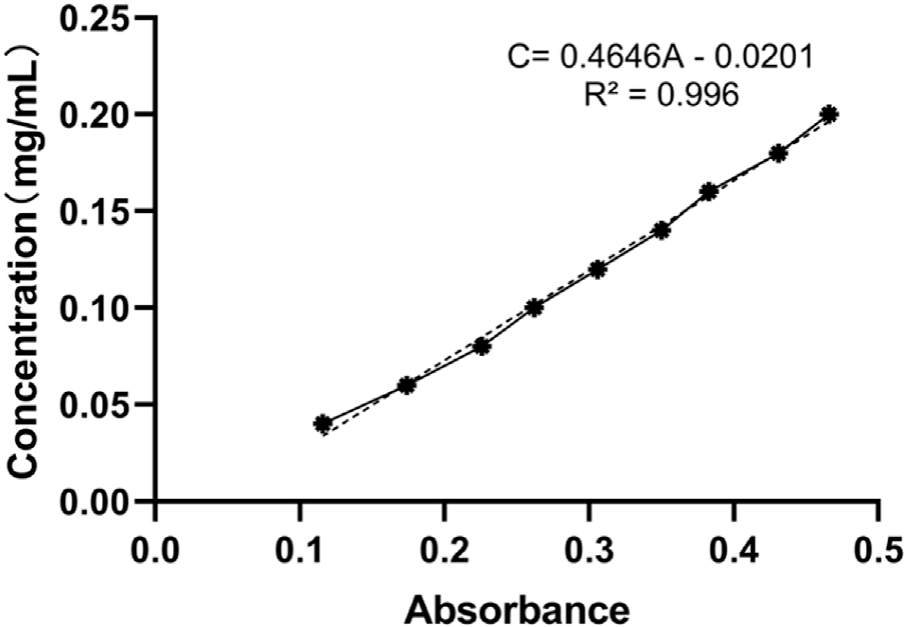
Solubility standard curve of Samoamide A.

In the water solubility experiment, the hydrophilic group was introduced into the original structure because of glycosylation modification, and we found that the water solubility of the product was significantly improved. After drawing and calculation, the linear regression equation of the standard curve was determined to be C = 0.4646A - 0.0201 (Figure 10). Then, we measured the absorbance value after dilution and calculated that the solubility increased from the original 0.73 mg/ml to the present 34.14 mg/ml. Representative solubility test data are shown, and complete data can be found in the Supplementary Materials.

## Conclusion

To sum up, we designed and synthesized a series of Samoamide A derivatives, including eight cyclic peptide derivatives scanned by arginine and glycosylation of arginine to modify inactive sites. Although it failed to show better antitumor activity, its water solubility was greatly improved. However, it is worth noting that we provide a general method of arginine N-glycosylation to modify cyclic peptides, using silver to promote solid-phase glycosylation; the subsequent use of this method can also replace glycosyl donors of glycosyl cyclic peptides, such as galactose, glucose, and rhamnose, to explore whether different glycosyl donors are related to their activity. This method provides a modification strategy for improving the properties of peptides. It is believed that with the gradual deepening of research, polypeptide drugs will have brighter application prospects after improving their properties.

## Data Availability

The datasets presented in this study can be found in online repositories. The names of the repository/repositories and accession number(s) can be found in the article/Supplementary Material.
