# Supplementary material for: Silver-promoted solid-phase guanidinylation enables the first synthesis of arginine glycosylated Samoamide A cyclopeptide analogue
Source: Front Chem. 2023 Jan 4;10:1040216. doi: 10.3389/fchem.2022.1040216 (PMC9846560; doi:10.3389/fchem.2022.1040216)
Supplement: Supplementary file 1 [file DataSheet1.PDF]

Supplementary Material

A

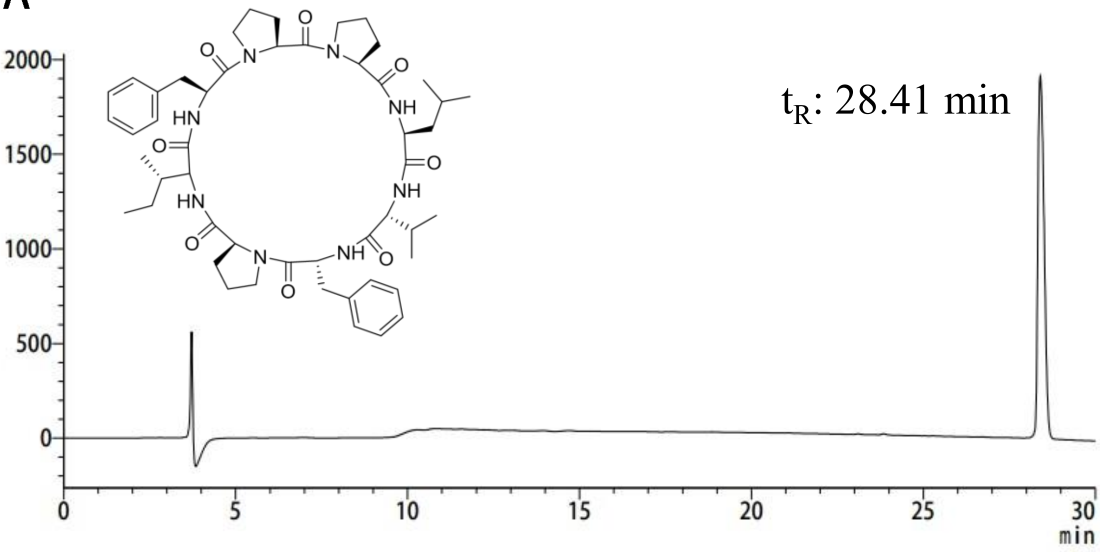

B

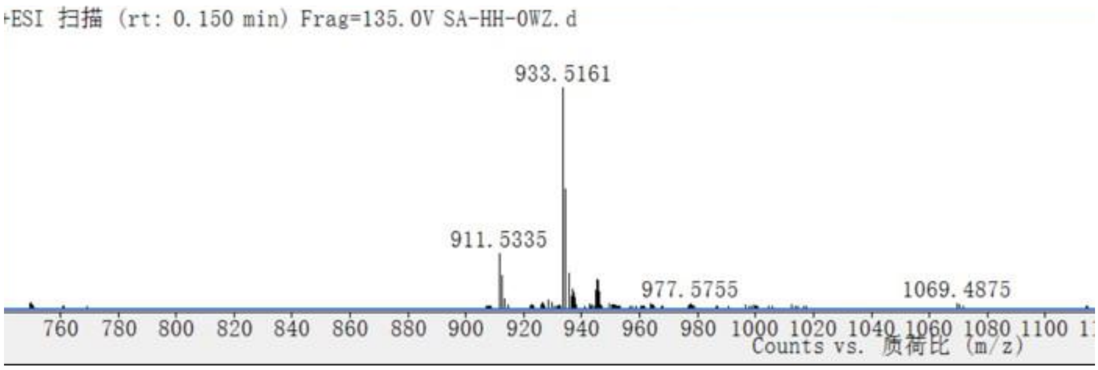

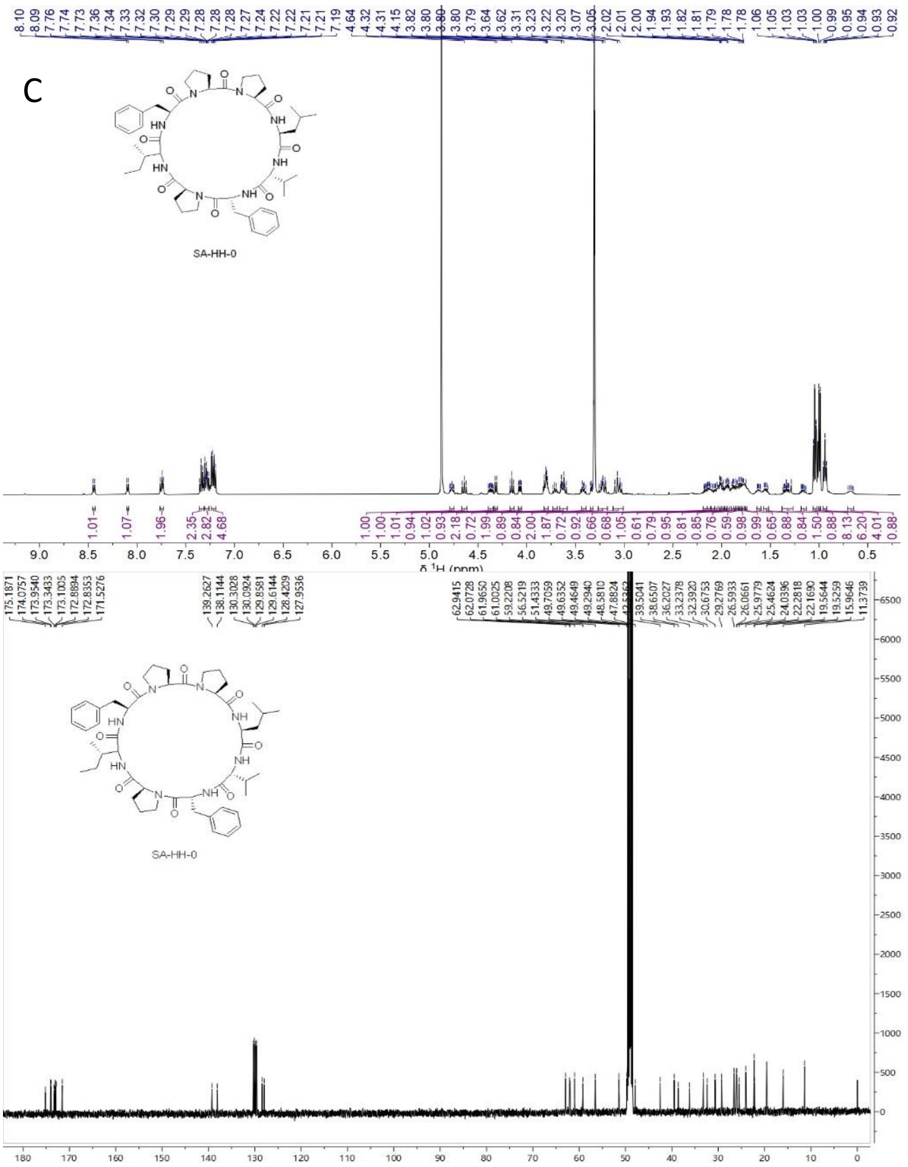

Supplementary Figure 1 A) The HPLC of purified SA-HH-0; **B)** ESI-MS spectrum of SA-HH-0 ESI-MS m/z calcd. For $C_{50}H_{70}N_8O_8$ 910.53; found $[M + H]^+$ = 911.53; $[M + Na]^+$ =933.51.**C)** The NMR spectra of SA-HH-0. ^1H NMR (500 MHz, Methanol-*d*$_4$) δ 8.44 (d, *J* = 8.5 Hz, 1H), 8.10 (d, *J* = 7.6 Hz, 1H), 7.74 (t, *J* = 7.6 Hz, 2H), 7.36 – 7.31 (m, 3H), 7.30 – 7.26 (m, 3H), 7.22 (dt, *J* = 8.3, 6.7 Hz, 5H), 4.79 – 4.75 (m, 1H), 4.64 (t, *J* = 10.3 Hz, 1H), 4.39 – 4.34 (m, 1H), 4.32 (d, *J* = 7.8 Hz, 1H), 4.15 (t, *J* = 8.7 Hz, 1H), 4.09 – 4.05 (m, 1H), 3.82 – 3.78 (m, 2H), 3.73 – 3.69 (m, 1H), 3.66 – 3.58 (m, 2H), 3.44 – 3.39 (m, 1H), 3.35 – 3.32 (m, 1H), 3.27 – 3.17 (m, 2H), 3.11 – 3.01 (m, 2H), 2.19 – 2.15 (m, 1H), 2.15 – 2.11 (m, 1H), 2.11 – 2.07 (m, 1H), 2.07 – 2.04 (m, 1H), 2.03 – 2.01 (m, 1H), 2.00 – 1.97 (m, 1H), 1.96 (s, 1H), 1.94 – 1.92 (m, 1H), 1.90 – 1.88 (m, 1H), 1.88 – 1.85 (m, 1H), 1.84 (d, *J* = 2.5 Hz, 1H), 1.82 (s, 1H), 1.81 – 1.79 (m, 1H), 1.79 – 1.77 (m, 1H), 1.75 (s, 1H), 1.64 – 1.60 (m, 1H), 1.57 – 1.52 (m, 1H), 1.38 – 1.27 (m, 1H), 1.18 – 1.13 (m, 1H), 1.06 – 1.02 (m, 8H), 0.99 (d, *J* = 6.6 Hz, 6H), 0.96 – 0.92 (m, 4H), 0.71 – 0.64 (m, 1H). ^{13}C NMR (126 MHz, MeOD) δ 175.18, 174.06, 173.94, 173.33, 173.09, 172.88, 172.82, 171.52, 139.25, 138.10, 130.29, 130.08, 129.85, 129.60, 128.41, 127.94, 62.93, 62.06, 61.95, 60.99, 59.21, 56.51, 51.42, 49.69, 49.62, 49.45, 49.28, 49.15, 49.11, 48.57, 47.87, 42.52, 39.49, 38.64, 36.19, 33.23, 32.38, 30.66, 29.27, 26.58, 26.05, 25.97, 25.45, 24.03, 22.27, 22.16, 19.55, 19.51, 15.95, 11.36.

A

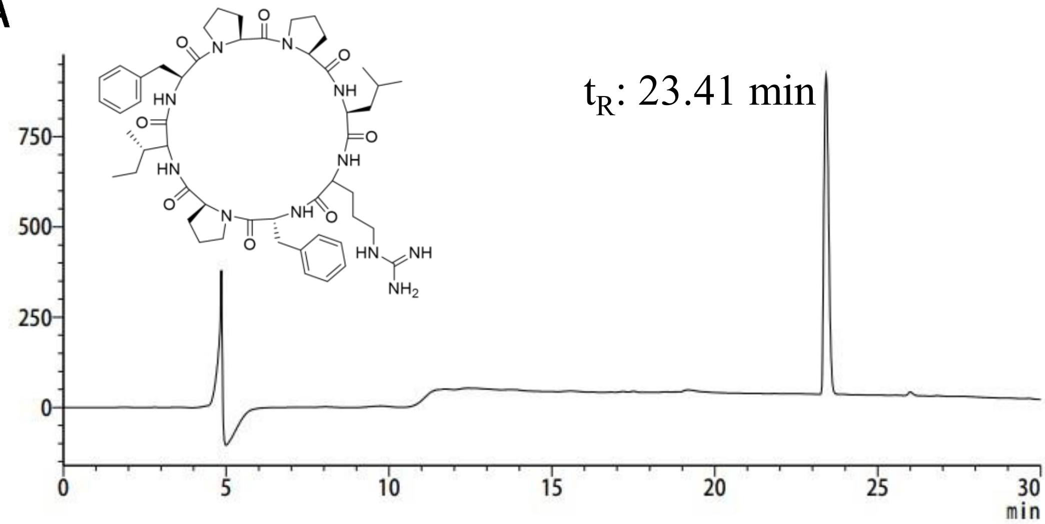
tR: 23.41 min
750
500
250
0
0
5
10
15
20
25
30
min

B

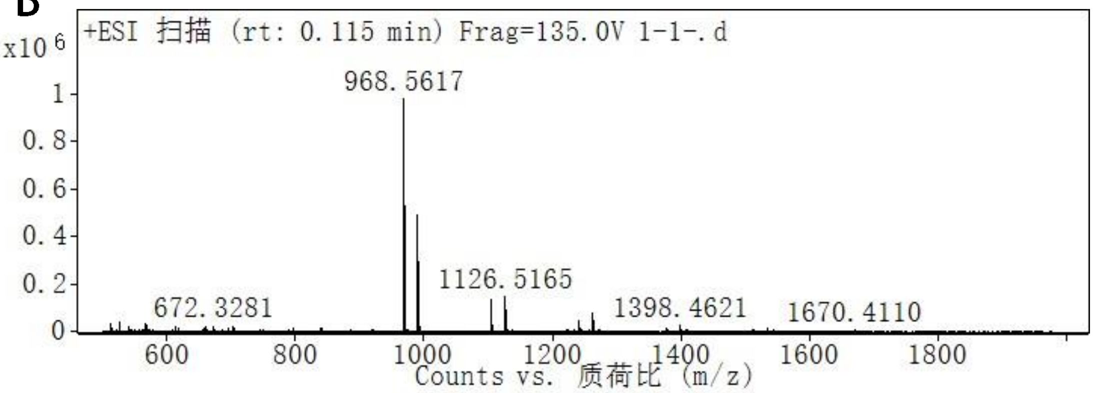
+ESI 扫描 (rt: 0.115 min) Frag=135.0V 1-1-.d
x10 6
968.5617
1126.5165
672.3281
1398.4621
1670.4110
Counts vs. 质荷比 (m/z)

C

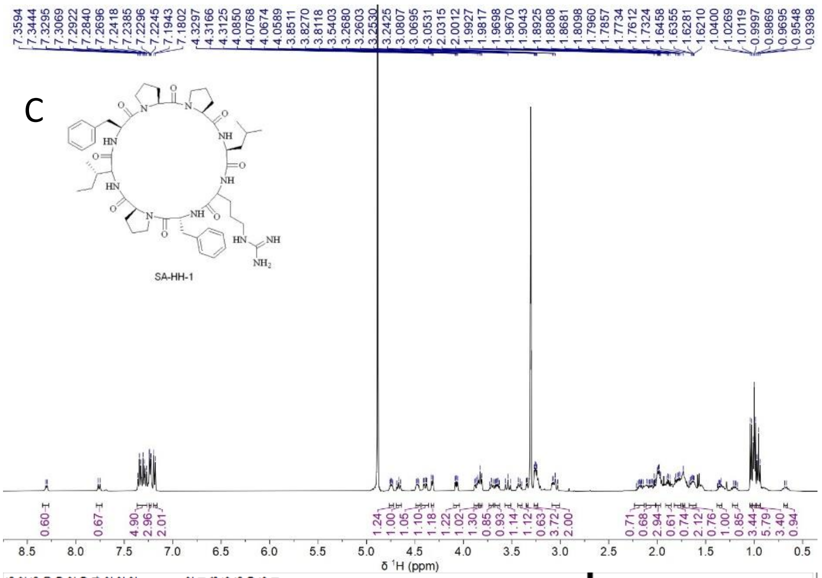

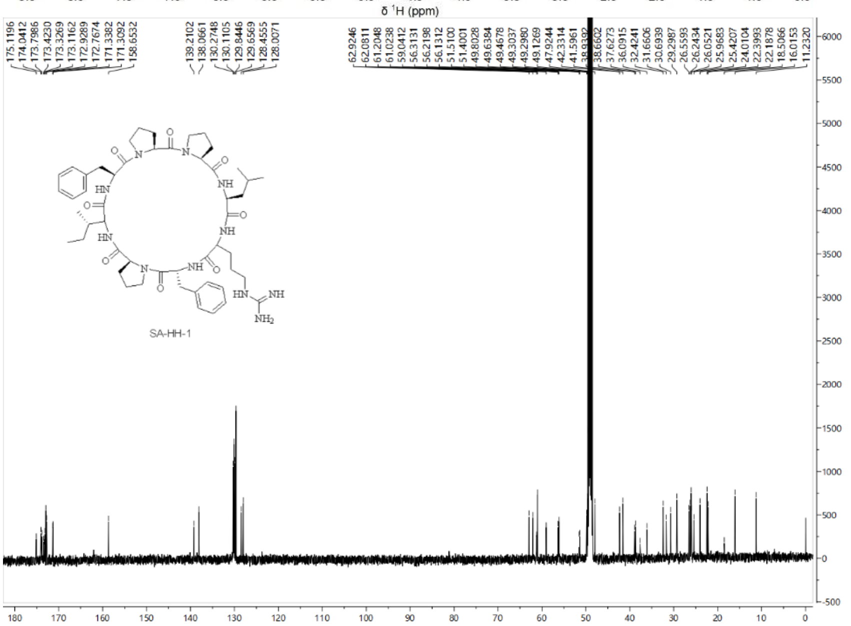

Supplementary Figure 2 A) The HPLC of purified SA-HH-1; **B)** ESI-MS spectrum of SA-HH-1 ESI-MS m/z calcd. For $C_{51}H_{73}N_{11}O_8$ 967.56; found $[M + H]^+$ = 968.56. **C)** The NMR spectra of SA-HH-1. ^{1}H NMR (500 MHz, Methanol-d_4) δ 8.30 (d, J = 7.3 Hz, 1H), 7.76 (d, J = 9.8 Hz, 1H), 7.37 – 7.27 (m, 5H), 7.24 – 7.22 (m, 3H), 7.19 (d, J = 7.0 Hz, 2H), 4.76 – 4.72 (m, 1H), 4.67 (t, J = 9.8 Hz, 1H), 4.49 – 4.44 (m, 1H), 4.43 – 4.36 (m, 1H), 4.33 – 4.31 (m, 1H), 4.10 – 4.04 (m, 1H), 3.89 – 3.85 (m, 1H), 3.82 (d, J = 7.6 Hz, 1H), 3.71 (t, J = 8.6 Hz, 1H), 3.68 – 3.63 (m, 1H), 3.54 (t, J = 12.7 Hz, 1H), 3.44 – 3.39 (m, 1H), 3.35 (d, J = 4.3 Hz, 1H), 3.27 – 3.23 (m, 4H), 3.09 – 3.01 (m, 2H), 2.24 – 2.13 (m, 1H), 2.12 – 2.02 (m, 1H), 2.01 – 1.97 (m, 3H), 1.91 – 1.86 (m, 1H), 1.82 – 1.75 (m, 1H), 1.73 (s, 2H), 1.67 – 1.60 (m, 1H), 1.38 – 1.33 (m, 1H), 1.22 – 1.17 (m, 1H), 1.03 (d, J = 6.6 Hz, 3H), 1.00 (t, J = 6.3 Hz, 6H), 0.95 (t, J = 7.4 Hz, 3H), 0.69 – 0.66 (m, 1H). ^{13}C NMR (126 MHz, Methanol-d_4) δ 175.12 , 174.04 , 173.80 , 173.42 , 173.33 , 173.12 , 172.93 , 172.77 , 171.34 , 171.31 , 158.65 , 139.21 , 138.07 , 130.27 , 130.11 , 129.84 , 129.66 , 128.46 , 128.01 , 62.92 , 62.08 , 61.20 , 61.02 , 59.04 , 56.31 , 56.22 , 56.13 , 51.51 , 51.40 , 49.80 , 47.92 , 42.33 , 41.60 , 38.94 , 38.66 , 36.09 , 32.42 , 31.66 , 30.69 , 29.30 , 26.56 , 26.24 , 26.05 , 25.97 , 25.42 , 24.01 , 22.40 , 22.19 , 18.51, 16.02 , 11.23 .

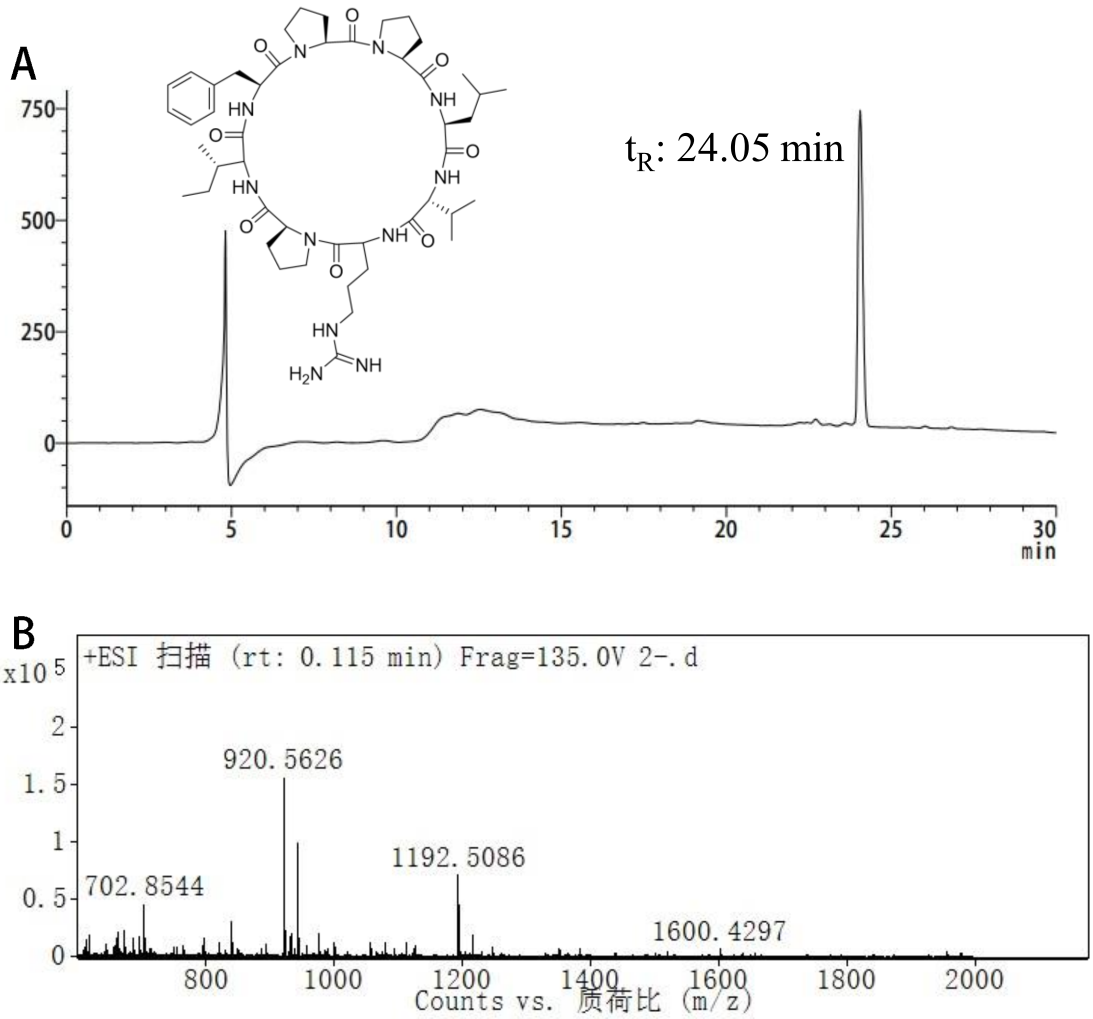
A
t_R: 24.05 min
750
500
250
0
0
5
10
15
20
25
30
min
B
+ESI 扫描 (rt: 0.115 min) Frag=135.0V 2-.d
x10 5
2
1.5
1
0.5
0
920.5626
1192.5086
702.8544
1600.4297
800
1000
1200
1400
1600
1800
2000
Counts vs. 质荷比 (m/z)

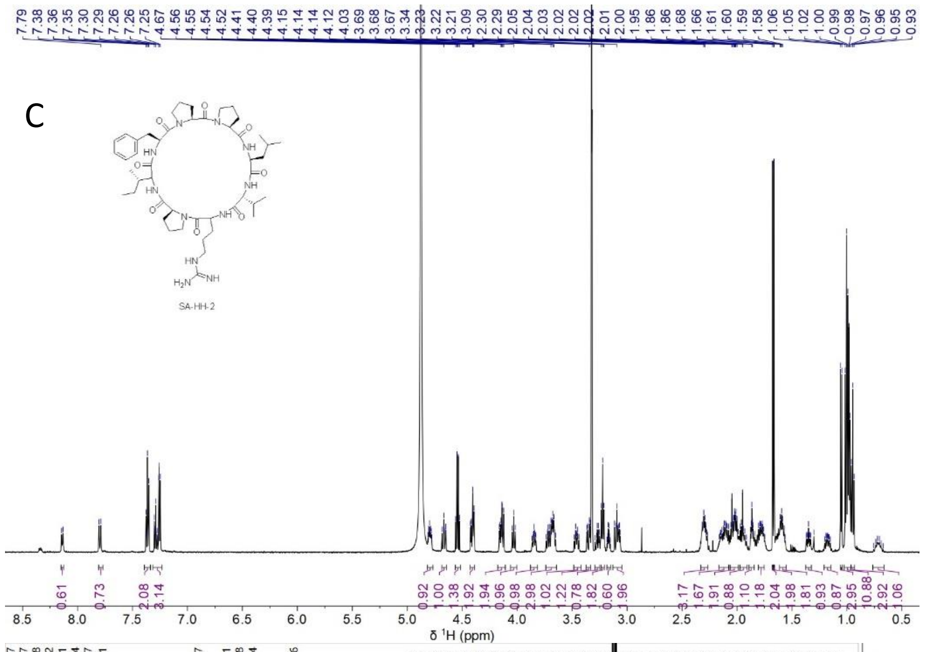

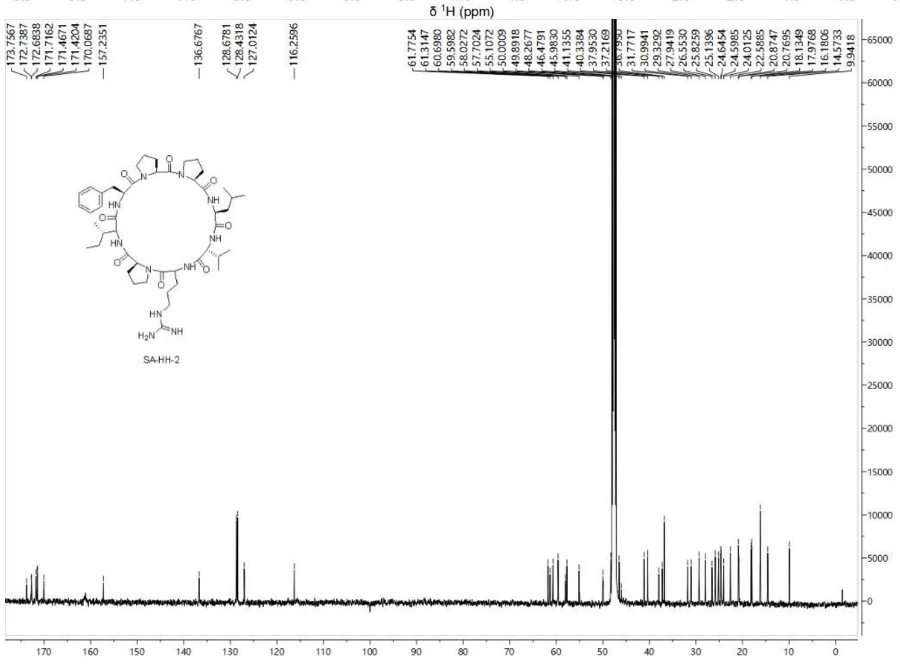

Supplementary Figure 3 A) The HPLC of purified SA-HH-2; **B)** ESI-MS spectrum of SA-HH-2 ESI-MS m/z calcd. For $C_{47}H_{73}N_{11}O_8$ 919.56; found [M + H] $^+$ = 920.56. **C)** The NMR spectra of SA-HH-2. ^{1}H NMR (600 MHz, Methanol-d_4) δ 8.14 (d, *J* = 7.6 Hz, 1H), 7.80 (d, *J* = 9.7 Hz, 1H), 7.39 – 7.34 (m, 2H), 7.32 – 7.23 (m, 3H), 4.82 – 4.77 (m, 1H), 4.69 – 4.64 (m, 1H), 4.54 (q, *J* = 7.0 Hz, 1H), 4.43 – 4.39 (m, 2H), 4.18 – 4.11 (m, 2H), 4.03 (t, *J* = 8.0 Hz, 1H), 3.88 – 3.81 (m, 1H), 3.74 – 3.64 (m, 3H), 3.49 – 3.42 (m, 1H), 3.37 – 3.33 (m, 1H), 3.29 – 3.25 (m, 1H), 3.22 (t, *J* = 7.0 Hz, 2H), 3.18 – 3.15 (m, 1H), 3.13 – 3.05 (m, 2H), 2.33 – 2.26 (m, 3H), 2.17 – 2.07 (m, 2H), 2.06 – 1.99 (m, 2H), 1.97 – 1.91 (m, 1H), 1.89 – 1.84 (m, 1H), 1.80 – 1.75 (m, 1H), 1.68 (s, 2H), 1.66 (s, 2H), 1.61 – 1.55 (m, 2H), 1.37 – 1.32 (m, 1H), 1.21 – 1.15 (m, 1H), 1.05 (d, *J* = 6.6 Hz, 3H), 1.02 – 0.96 (m, 11H), 0.95 (t, *J* = 7.4 Hz, 3H), 0.76 – 0.66 (m, 1H). ^{13}C NMR (151 MHz, Methanol-d_4) δ 173.76 , 172.74 , 172.68 , 171.72 , 171.44, 170.07 , 157.24 , 136.68 , 128.68 , 128.43 , 127.01 , 116.26 , 61.78 , 61.31 , 60.70 , 59.60 , 58.03 , 57.70 , 55.11 , 50.00 , 49.89 ,

48.27 , 46.48 , 45.98 , 41.14 , 40.34 , 37.95 , 37.22 , 36.80 , 31.77 , 30.99 , 29.33 , 27.94 , 26.55 , 25.83 , 25.14 , 24.65 , 24.60 , 24.01 , 22.59 , 20.87 , 20.77 , 18.13 , 17.98 , 16.18 , 14.57 , 9.94 .

A

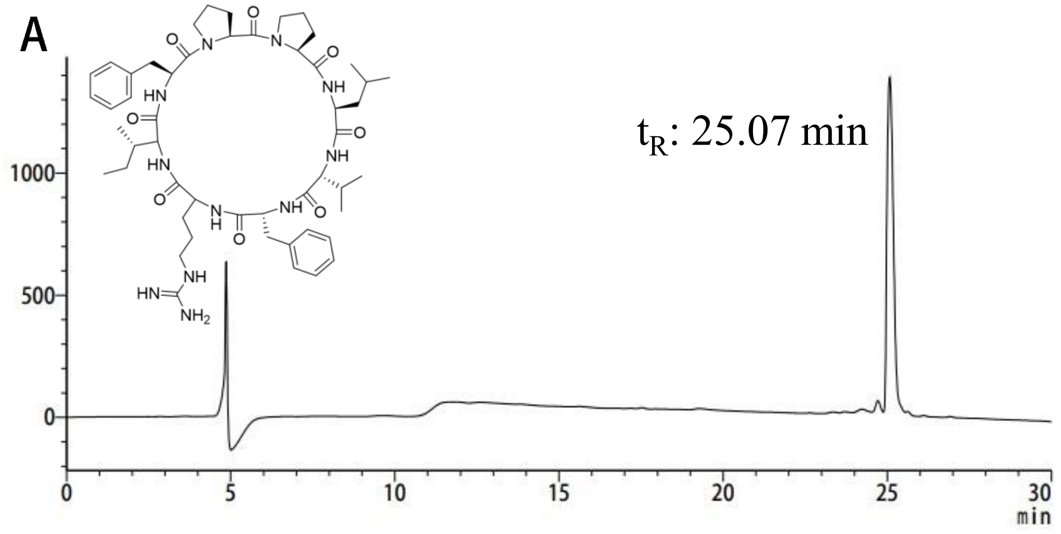

B

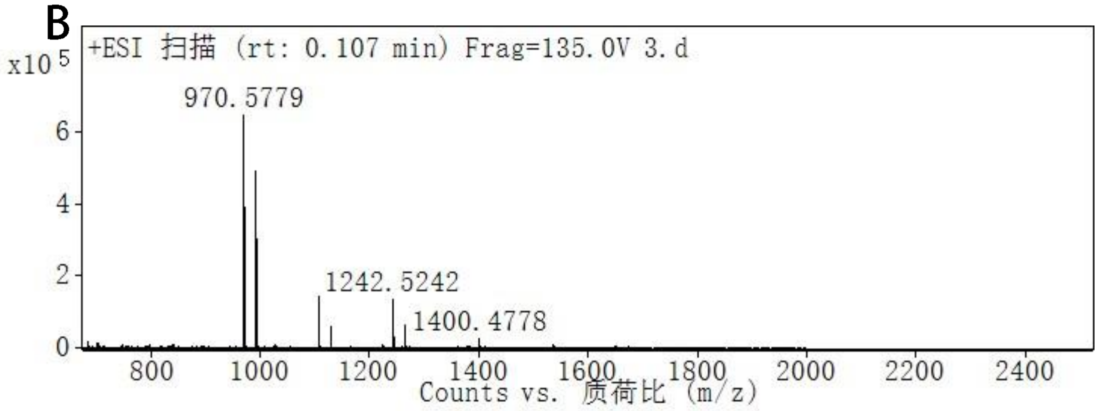

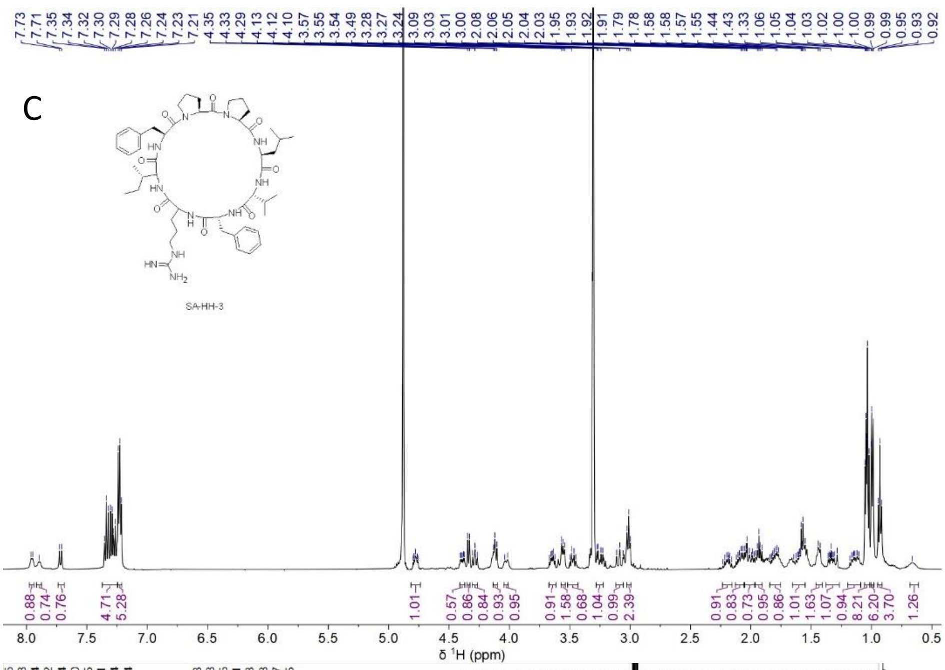

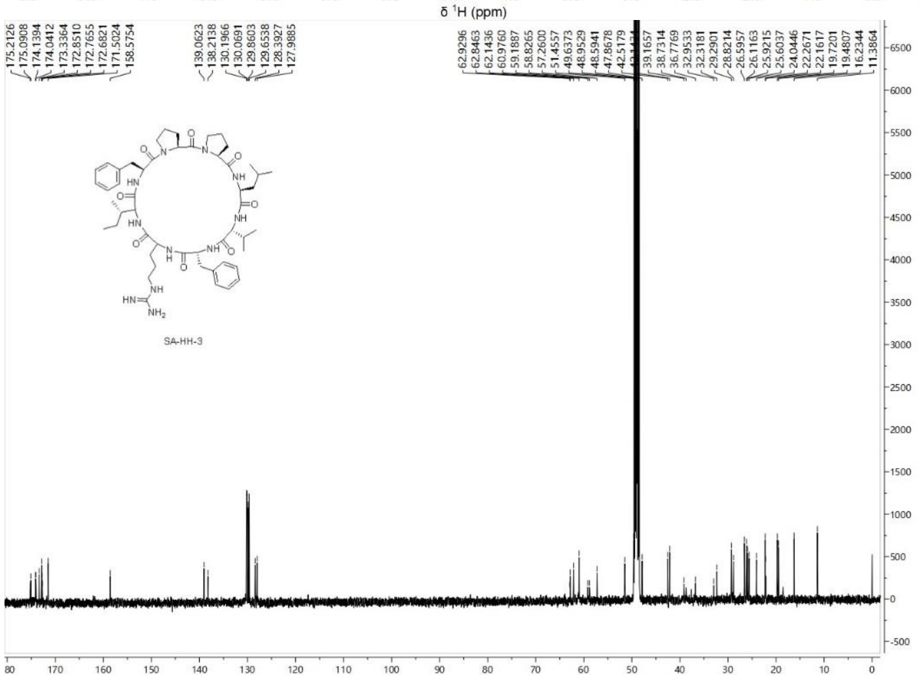

Supplementary Figure 4 A) The HPLC of purified SA-HH-3; **B)** ESI-MS spectrum of SA-HH-3 ESI-MS m/z calcd. For $C_{51}H_{75}N_{11}O_8$ 969.568; found $[M + H]^+$ = 970.57. **C)** The NMR spectra of SA-HH-3. ^{1}H NMR (500 MHz, Methanol-d_4) δ 7.96 (d, J = 7.6 Hz, 1H), 7.89 (s, 1H), 7.72 (d, J = 9.5 Hz, 1H), 7.37 – 7.25 (m, 5H), 7.25 – 7.21 (m, 5H), 4.82 – 4.74 (m, 1H), 4.41 – 4.37 (m, 1H), 4.34 (d, J = 8.1 Hz, 1H), 4.29 (t, J = 9.7 Hz, 1H), 4.12 (t, J = 6.8 Hz, 1H), 4.03 (d, J = 11.9 Hz, 1H), 3.67 – 3.61 (m, 1H), 3.57 – 3.54 (m, 2H), 3.52 – 3.43 (m, 1H), 3.28 – 3.22 (m, 1H), 3.12 – 3.06 (m, 1H), 3.01 (t, J = 6.8 Hz, 2H), 2.23 – 2.16 (m, 1H), 2.13 – 2.06 (m, 1H), 2.05 – 1.97 (m, 1H), 1.96 – 1.90 (m, 1H), 1.84 – 1.75 (m, 1H), 1.66 – 1.55 (m, 1H), 1.43 (d, J = 7.7 Hz, 2H), 1.38 – 1.27 (m, 1H), 1.20 – 1.09 (m, 1H), 1.06 – 1.02 (m, 8H), 1.01 – 0.98 (m, 6H), 0.93 (t, J = 7.3 Hz, 4H), 0.66 (s, 1H). ^{13}C NMR (126 MHz, MeOD) δ 175.21, 175.09, 174.14, 174.04, 173.34, 172.85, 172.77, 172.68, 171.50, 158.58, 139.06, 138.21, 130.20, 130.07, 129.86, 129.65, 128.39, 127.99, 62.93, 62.85, 62.14, 60.98, 59.19, 58.83, 57.26, 51.46, 49.64, 48.95, 48.59, 47.87, 42.52, 42.14, 39.17,

38.73, 36.78, 32.95, 32.32, 29.29, 28.82, 26.60, 26.12, 25.92, 25.60, 24.04, 22.27, 22.16, 19.72, 19.48, 16.23, 11.39.

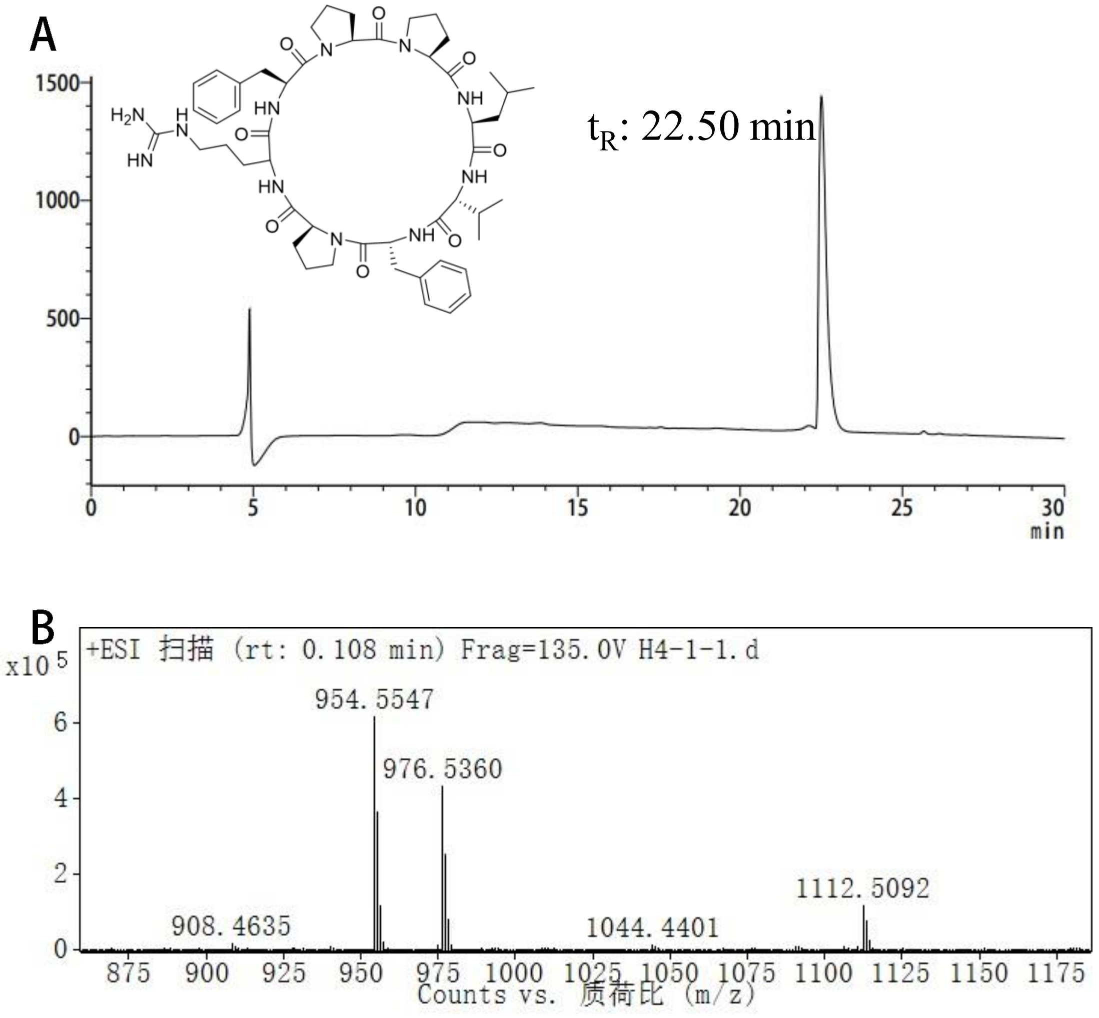

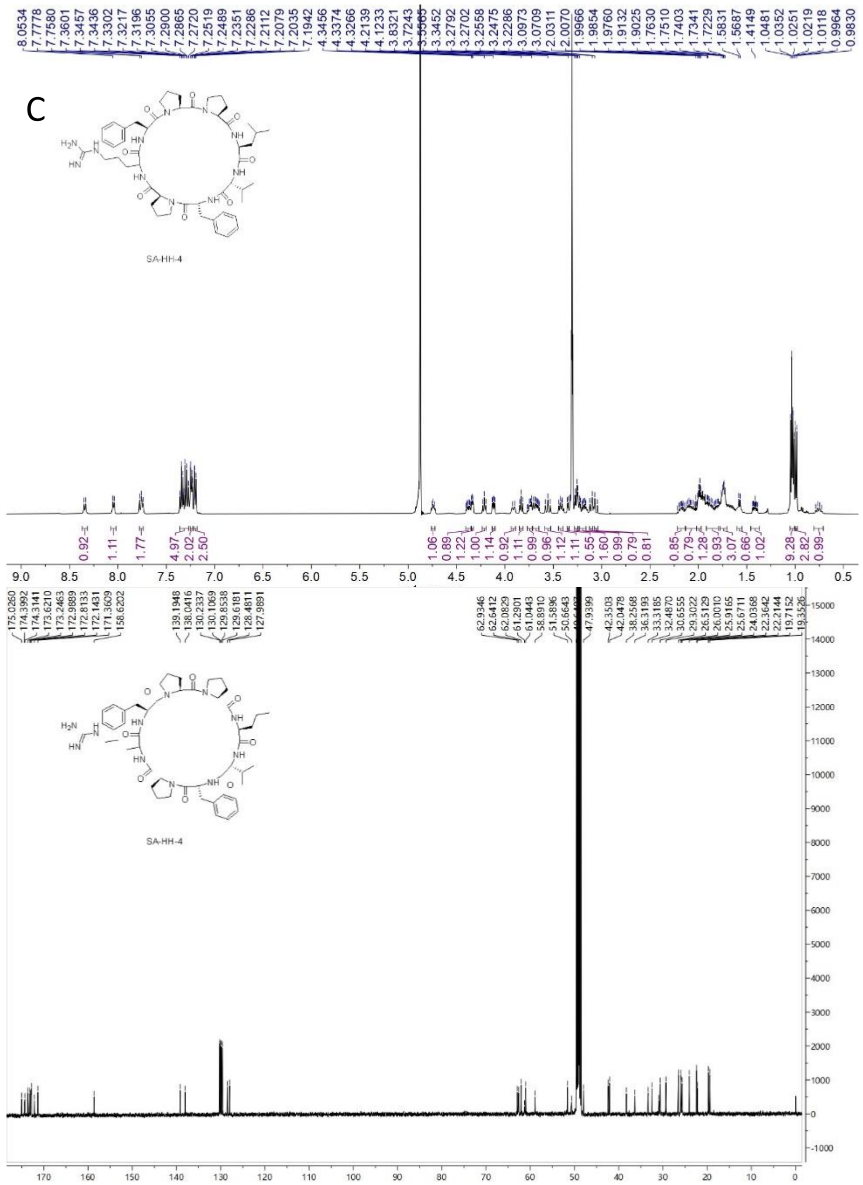

Supplementary Figure 5 A) The HPLC of purified SA-HH-4; **B)** ESI-MS spectrum of SA-HH-4 ESI-MS m/z calcd. For $C_{50}H_{71}N_{11}O_8$ 953.53; found $[M + H]^+$ = 954.55. **C)** The NMR spectra of SA-HH-4. ^{1}H NMR (500 MHz, Methanol-d_4) δ 8.34 (d, J = 8.7 Hz, 1H), 8.05 (d, J = 7.3 Hz, 1H), 7.78 – 7.74 (m, 2H), 7.36 – 7.27 (m, 5H), 7.25 – 7.23 (m, 2H), 7.21 – 7.18 (m, 2H), 4.76 – 4.72 (m, 1H), 4.41 – 4.35 (m, 1H), 4.34 (t, J = 4.8 Hz, 1H), 4.21 (t, J = 8.5 Hz, 1H), 4.13 – 4.10 (m, 1H), 3.91 (d, J = 12.0 Hz, 1H), 3.83 (t, J = 7.5 Hz, 1H), 3.77 – 3.72 (m, 1H), 3.71 – 3.65 (m, 1H), 3.56 (t, J = 12.8 Hz, 1H), 3.45 – 3.40 (m, 1H), 3.35 (d, J = 4.5 Hz, 1H), 3.28 – 3.25 (m, 2H), 3.23 – 3.16 (m, 1H), 3.11 (d, J = 12.9 Hz, 1H), 3.06 (d, J = 13.2 Hz, 1H), 2.22 – 2.14 (m, 1H), 2.13 – 2.02 (m, 1H), 2.02 – 1.97 (m, 1H), 1.92 – 1.79 (m, 1H), 1.77 – 1.71 (m, 3H), 1.60 – 1.55 (m, 1H), 1.46 – 1.37 (m, 1H), 1.05 – 1.01 (m, 9H), 0.99 (d, J = 6.7 Hz, 3H), 0.81 – 0.71 (m, 1H). ^{13}C NMR (126 MHz, MeOD) δ 175.03, 174.40, 174.31, 173.62, 173.25, 172.99, 172.81, 172.14, 171.36, 158.62, 139.19, 138.04, 130.23, 130.11, 129.85, 129.62, 128.48, 127.99, 62.93, 62.64, 62.08, 61.29, 61.04, 58.89, 51.59, 50.66, 49.64, 49.47, 49.30, 49.19, 49.13, 47.94, 42.35, 42.05, 38.26, 36.32, 33.32, 32.49, 30.93, 30.66, 29.30, 26.51, 26.00, 25.92, 25.67, 24.04, 22.36, 22.21, 19.72, 19.35.

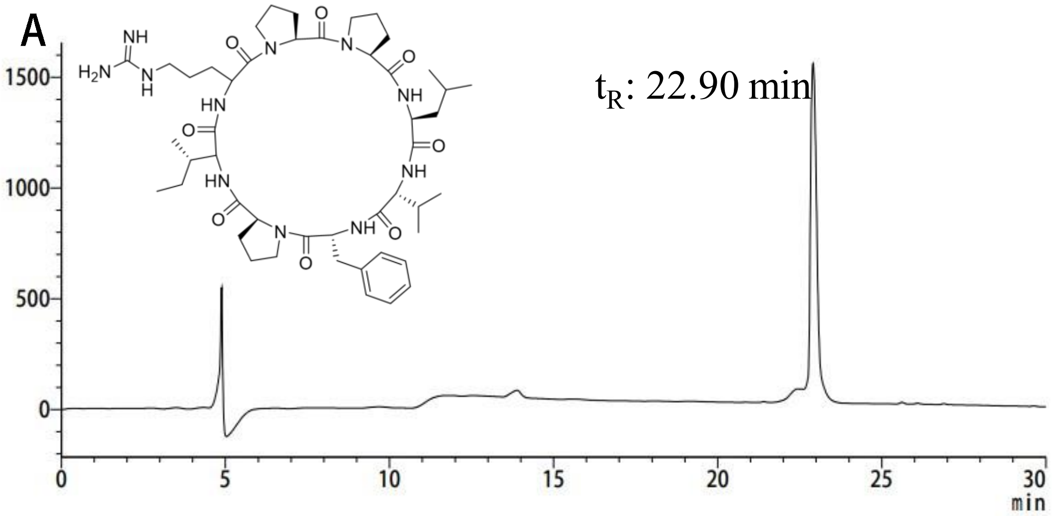
A
tR: 22.90 min
1500
1000
500
0
0
5
10
15
20
25
30
min

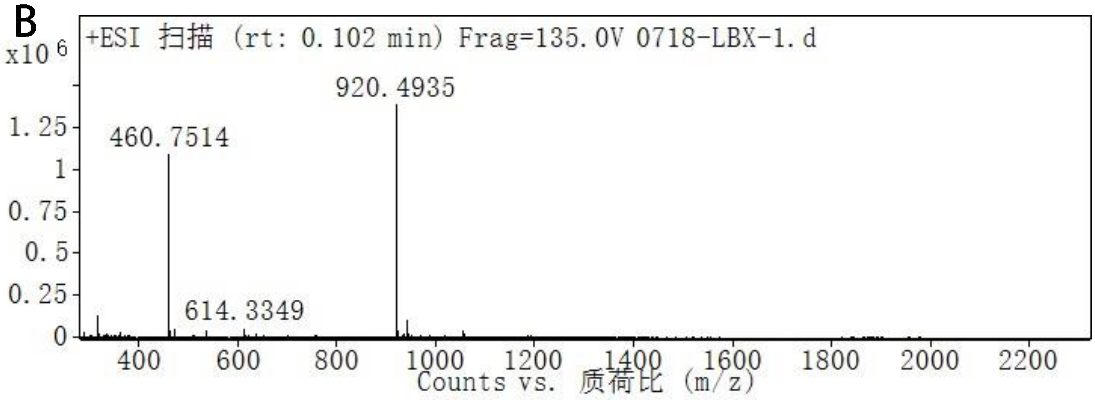
B
+ESI 扫描 (rt: 0.102 min) Frag=135.0V 0718-LBX-1.d
x10 6
920.4935
460.7514
614.3349
1.25
1
0.75
0.5
0.25
0
400
600
800
1000
1200
1400
1600
1800
2000
2200
Counts vs. 质荷比 (m/z)

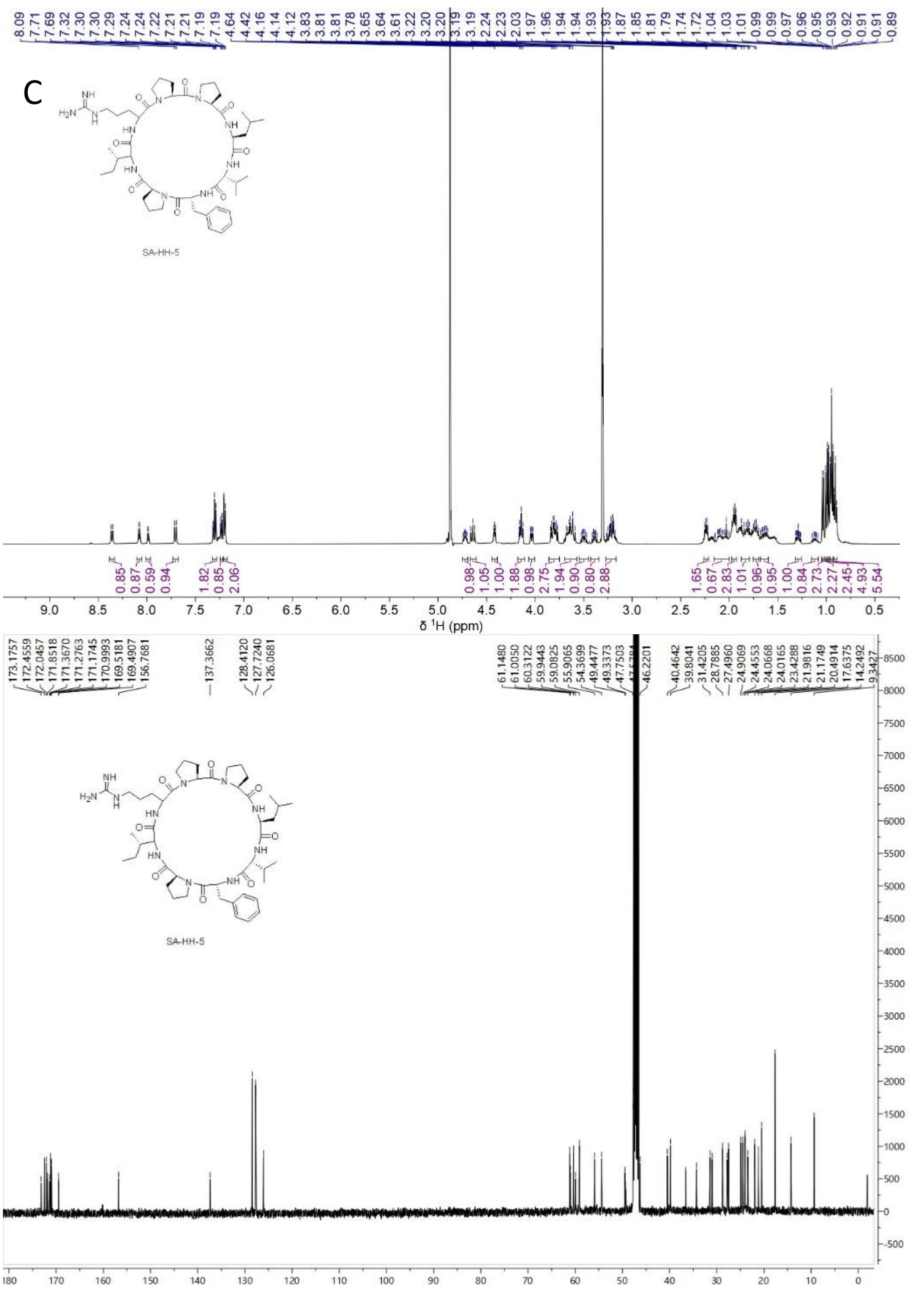

Supplementary Figure 6 A) The HPLC of purified SA-HH-5; **B)** ESI-MS spectrum of SA-HH-5 ESI-MS m/z calcd. For $C_{47}H_{73}N_{11}O_8$ 919.56; found [M + H] $^{+}$ = 920.49. **C)** The NMR spectra of SA-HH-5. ^{1}H NMR (500 MHz, Methanol-d_4) δ 8.36 (d, J = 8.6 Hz, 1H), 8.08 (d, J = 7.6 Hz, 1H), 7.99 (d, J = 6.3 Hz, 1H), 7.70 (d, J = 9.8 Hz, 1H), 7.32 – 7.28 (m, 2H), 7.24 – 7.22 (m, 1H), 7.20 (d, J = 8.2 Hz, 2H), 4.75 – 4.69 (m, 1H), 4.64 (t, J = 10.4 Hz, 1H), 4.42 (t, J = 5.0 Hz, 1H), 4.18 – 4.11 (m, 2H), 4.07 – 4.01 (m, 1H), 3.86 – 3.75 (m, 3H), 3.70 – 3.57 (m, 2H), 3.55 – 3.45 (m, 1H), 3.43 – 3.34 (m, 1H), 3.27 – 3.17 (m, 3H), 2.26 – 2.21 (m, 2H), 2.16 – 2.00 (m, 1H), 1.98 – 1.92 (m, 3H), 1.87 – 1.79 (m, 1H), 1.75 – 1.69 (m, 1H), 1.68 – 1.60 (m, 1H), 1.32 – 1.26 (m, 1H), 1.15 – 1.08 (m, 1H), 1.03 (d, J = 6.7 Hz, 3H), 1.00 (d, J = 6.8 Hz, 2H), 0.98 (d, J = 6.6 Hz, 2H), 0.95 (t, J = 6.4 Hz, 5H), 0.91 (t, J = 7.4 Hz, 6H). ^{13}C NMR (126 MHz, MeOD) δ 173.18, 172.46, 172.05, 171.85, 171.37, 171.28, 171.17, 171.00, 169.52, 169.49, 156.77, 137.37, 128.41, 127.72, 126.07, 61.15, 61.01, 60.31, 59.94, 59.08, 55.91, 54.37, 49.45, 49.34, 47.75, 47.58, 46.22, 40.46, 39.80, 36.57, 34.30, 31.42, 30.97, 28.79, 27.78, 27.50, 24.91, 24.46, 24.07, 24.02, 23.43, 21.98, 21.17, 20.49, 17.64, 14.25,

9.34.

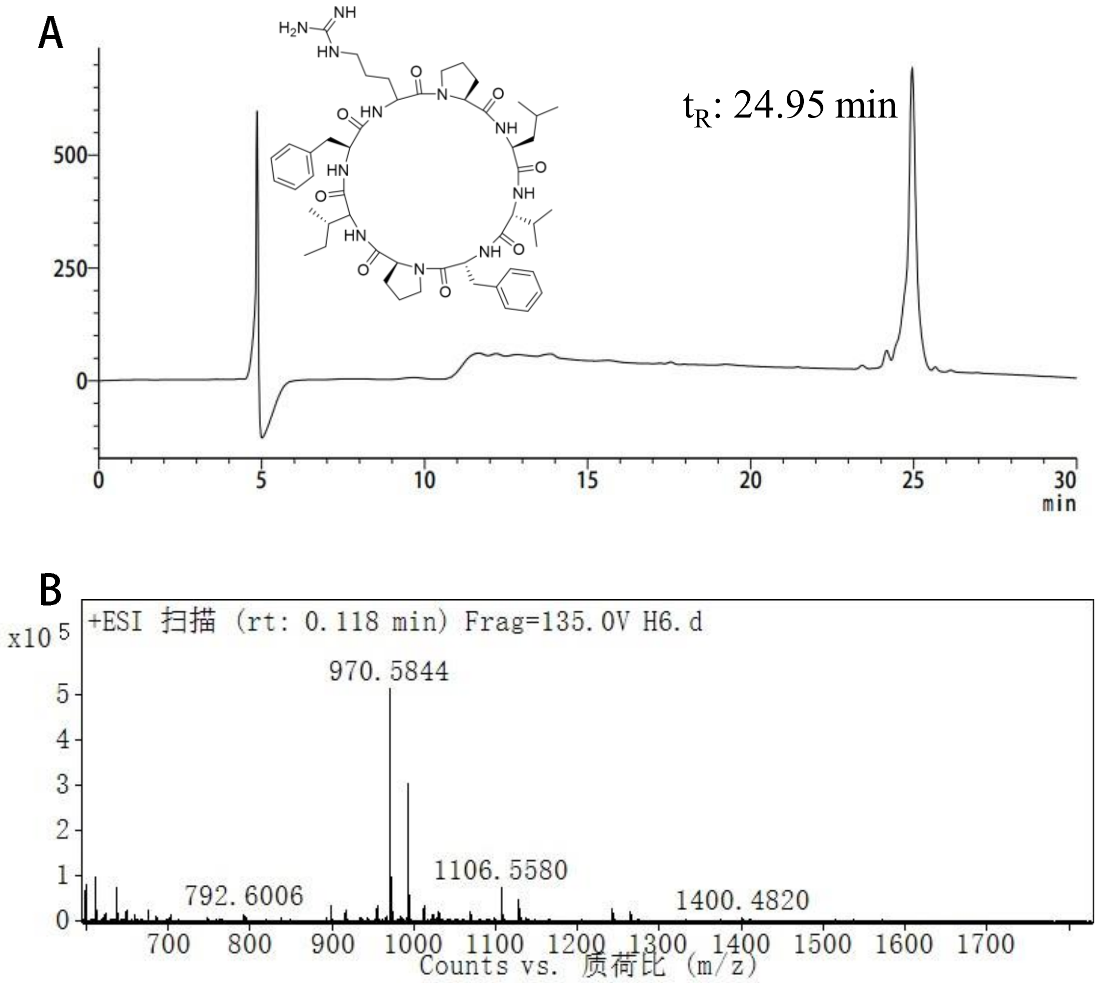

Supplementary Figure 7 **A)** The HPLC of purified SA-HH-6; **B)** ESI-MS spectrum of SA-HH-6 ESI-MS m/z calcd. For $C_{51}H_{75}N_{11}O_8$ 969.58; found $[M + H]^+$ = 970.58.

A

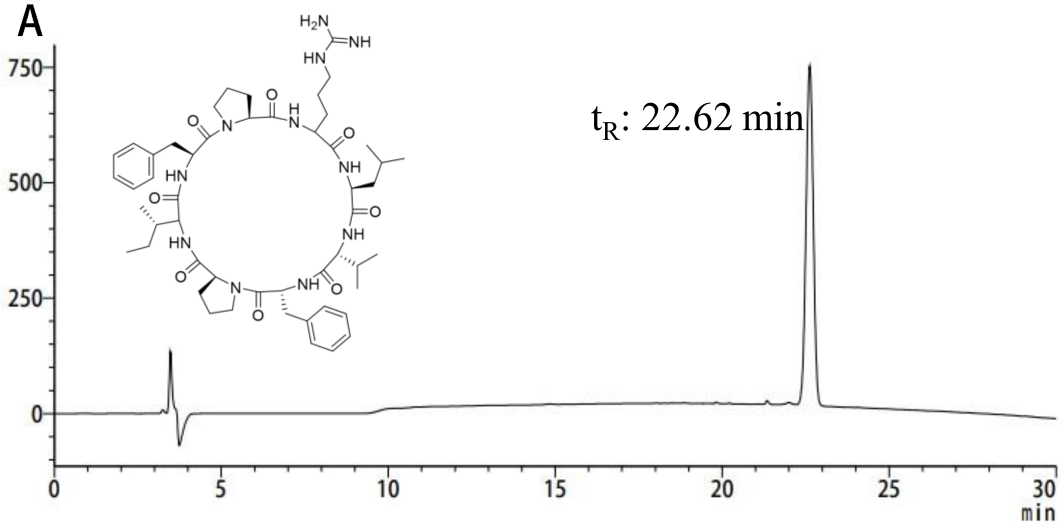

750
500
250
0
0
5
10
15
20
25
30
min
t_R: 22.62 min

B

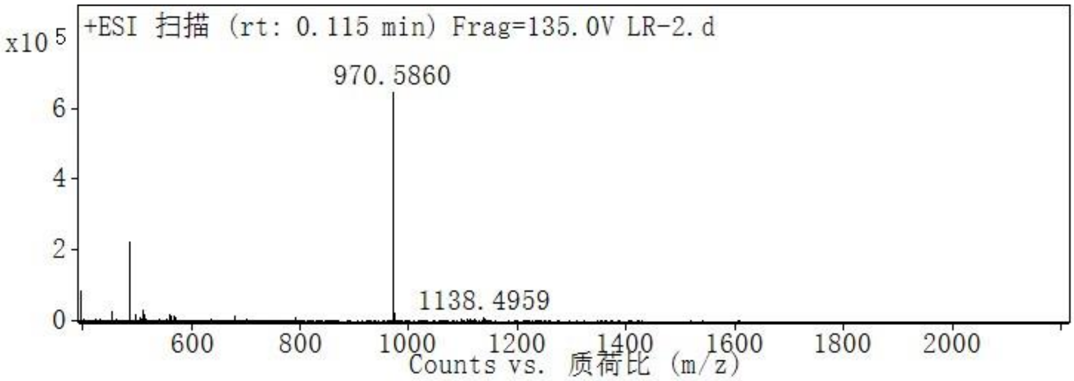

+ESI 扫描 (rt: 0.115 min) Frag=135.0V LR-2.d
x10 5
970.5860
1138.4959
Counts vs. 质荷比 (m/z)

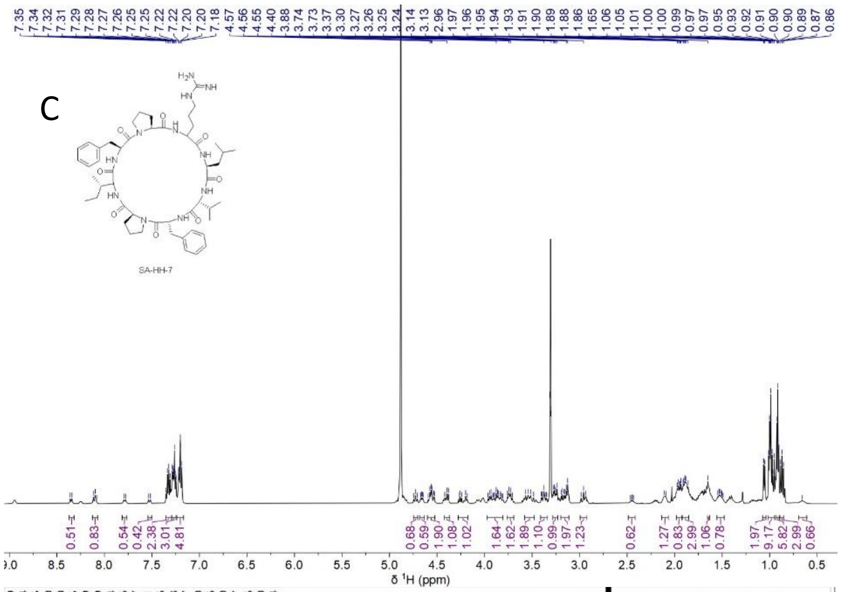

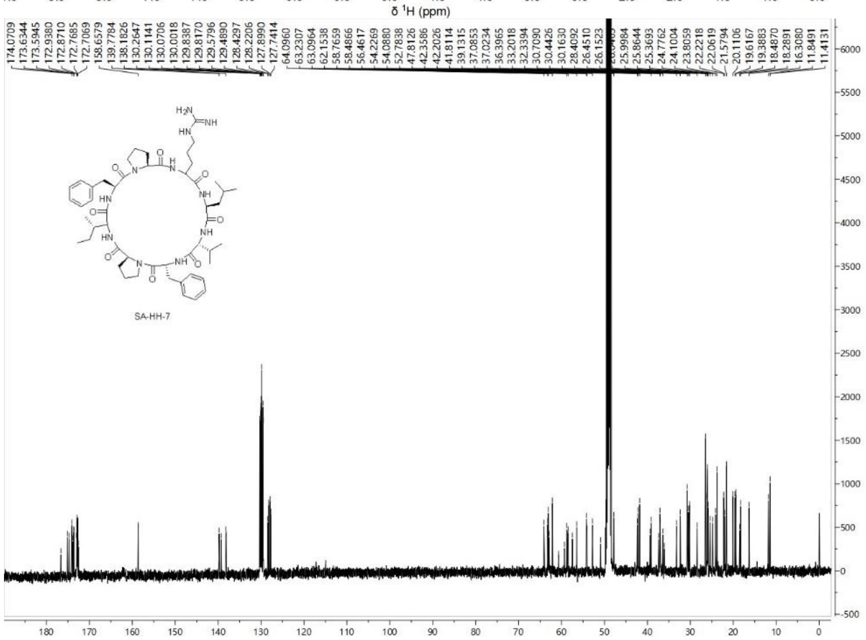

Supplementary Figure 8 A) The HPLC of purified SA-HH-7; **B)** ESI-MS spectrum of SA-HH-7 ESI-MS m/z calcd. For $C_{51}H_{75}N_{11}O_8$ 969.58; found $[M + H]^+$ = 970.58. **C)** The NMR spectra of SA-HH-7. ^1H NMR (500 MHz, Methanol-d_4) δ 8.35 (d, J = 9.3 Hz, 1H), 8.10 (t, J = 8.7 Hz, 1H), 7.79 (d, J = 9.4 Hz, 1H), 7.53 (d, J = 9.9 Hz, 1H), 7.35 – 7.30 (m, 2H), 7.29 – 7.25 (m, 3H), 7.24 – 7.17 (m, 5H), 4.73 (t, J = 9.9 Hz, 1H), 4.68 – 4.63 (m, 1H), 4.60 – 4.52 (m, 2H), 4.42 – 4.37 (m, 1H), 4.28 – 4.17 (m, 1H), 3.97 – 3.81 (m, 2H), 3.77 – 3.69 (m, 2H), 3.58 – 3.48 (m, 2H), 3.41 – 3.34 (m, 1H), 3.28 – 3.23 (m, 1H), 3.20 – 3.11 (m, 2H), 2.96 (t, J = 12.2 Hz, 1H), 2.49 – 2.42 (m, 1H), 2.10 (d, J = 9.2 Hz, 1H), 1.98 – 1.92 (m, 1H), 1.92 – 1.85 (m, 3H), 1.65 (s, 1H), 1.56 – 1.48 (m, 1H), 1.06 (d, J = 6.5 Hz, 2H), 1.02 – 0.95 (m, 9H), 0.93 – 0.90 (m, 6H), 0.89 – 0.85 (m, 3H), 0.66 (s, 1H). ^{13}C NMR (126 MHz, Methanol-d_4) δ 176.64 , 175.08 , 174.76 , 174.13 , 174.07 , 173.80 , 173.63 , 173.59 , 173.02 , 172.94 , 172.87 , 172.81 , 172.77 , 172.71 , 172.58 , 158.66 , 139.78 , 139.28 , 138.18 , 138.11 , 130.26 , 130.11 , 130.07 , 130.00 , 129.84 , 129.82 , 129.58 , 129.49 , 128.43 , 128.22 , 127.90 , 127.74 , 64.10 , 63.23 , 63.10 , 62.91 , 62.15 , 60.70 , 59.33 , 58.77 , 58.57 , 58.49 , 57.48 , 56.46 , 54.23 , 54.09 , 52.78 , 50.89 , 47.81 , 42.36 , 42.20 , 41.81 , 39.36 , 39.13 , 37.37 , 37.09 , 37.02 , 36.40 , 36.08 , 33.20 , 32.34 , 30.71 , 30.44 , 30.16 , 28.41 , 26.45 ,

26.15 , 26.05 , 26.00 , 25.86 , 25.37 , 24.78 , 24.10 , 23.81 , 22.22 , 22.06 , 21.58 , 20.11 , 19.62 , 19.39 , 18.49 , 18.29 , 16.31 , 11.85 , 11.41 .

A

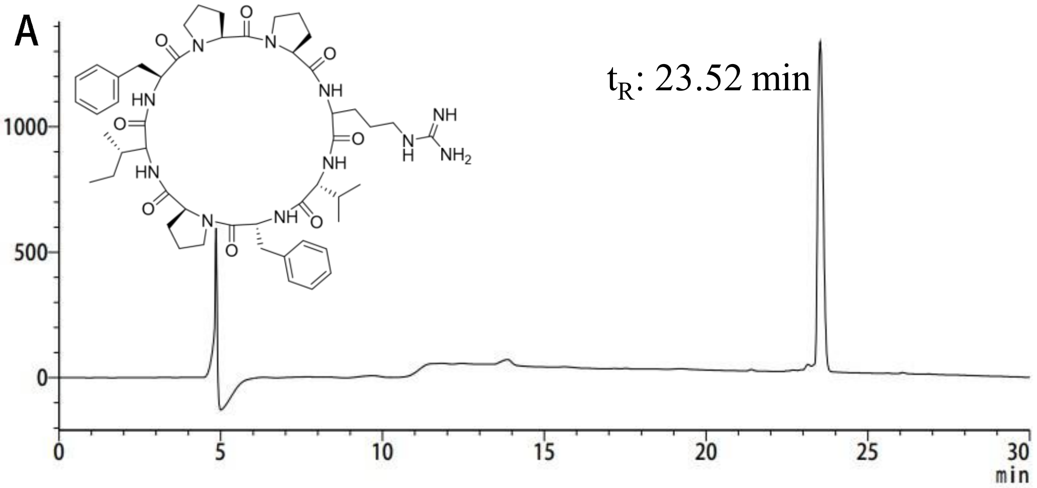

B

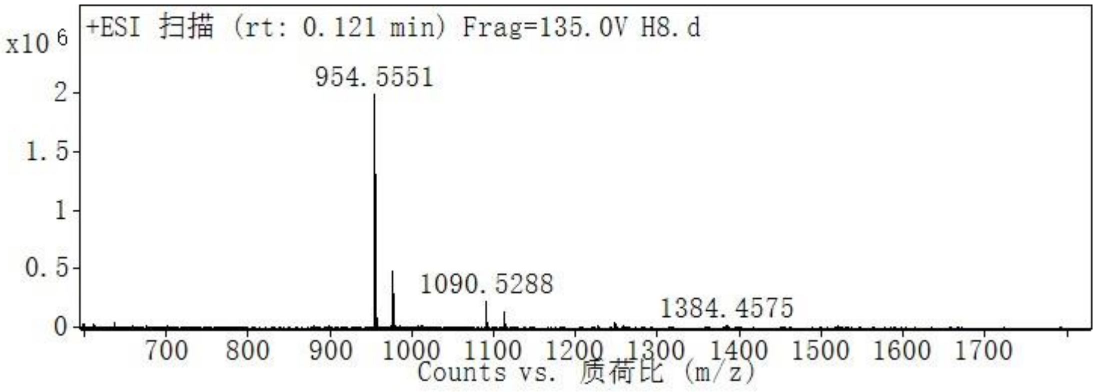

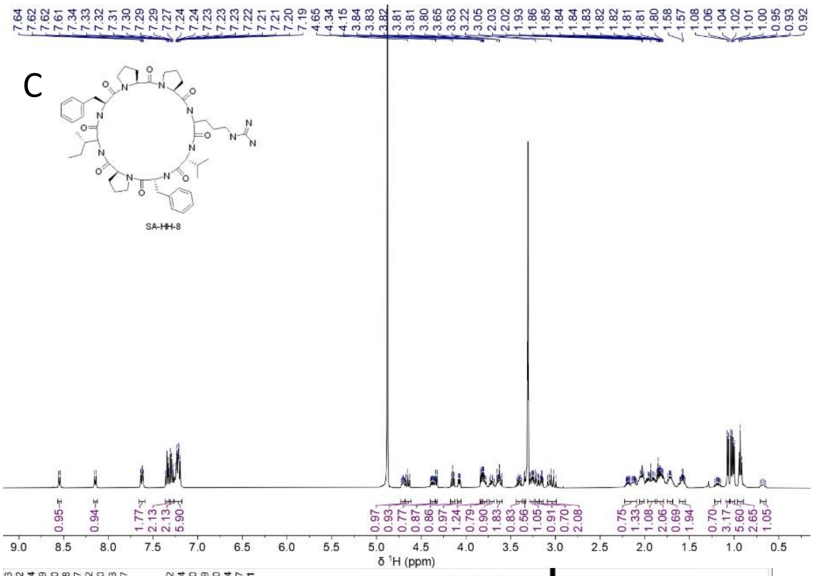

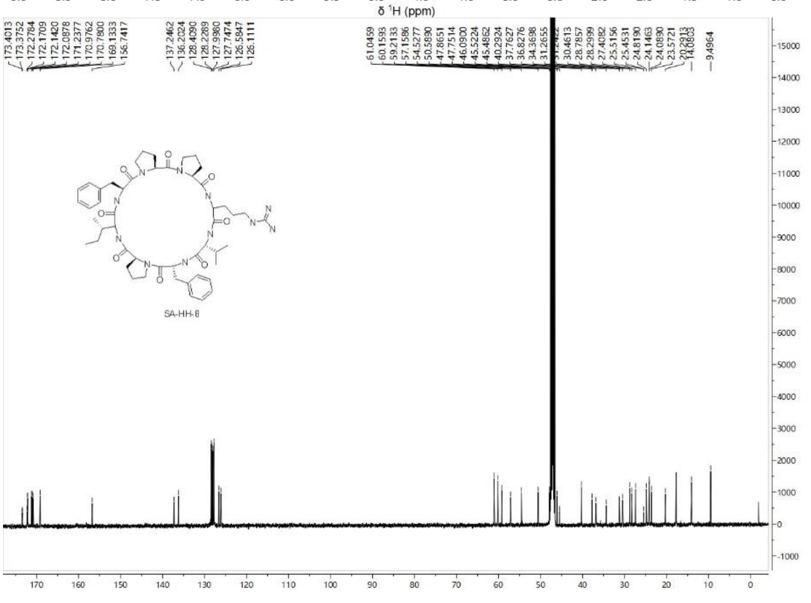

Supplementary Figure 9 A) The HPLC of purified SA-HH-8; **B)** ESI-MS spectrum of SA-HH-8 ESI-MS m/z calcd. For $C_{50}H_{71}N_{11}O_8$ 953.53; found $[M + H]^+$ = 954.55. **C)** The NMR spectra of SA-HH-8. ^{1}H NMR (500 MHz, Methanol-d_4) δ 8.55 (d, J = 8.3 Hz, 1H), 8.15 (d, J = 7.2 Hz, 1H), 7.62 (d, J = 8.3 Hz, 2H), 7.36 – 7.32 (m, 2H), 7.31 – 7.27 (m, 2H), 7.26 – 7.18 (m, 6H), 4.73 – 4.68 (m, 1H), 4.65 (t, J = 10.3 Hz, 1H), 4.40 – 4.35 (m, 1H), 4.33 (d, J = 7.7 Hz, 1H), 4.15 (t, J = 8.6 Hz, 1H), 4.07 (dd, J = 8.9, 4.0 Hz, 1H), 3.84 – 3.82 (m, 1H), 3.81 – 3.77 (m, 1H), 3.71 (q, J = 9.0, 8.6 Hz, 1H), 3.65 – 3.59 (m, 2H), 3.44 – 3.37 (m, 1H), 3.34 (d, J = 4.3 Hz, 1H), 3.29 – 3.24 (m, 1H), 3.22 – 3.18 (m, 1H), 3.17 – 3.13 (m, 1H), 3.09 – 2.99 (m, 2H), 2.23 – 2.09 (m, 1H), 2.06 – 2.01 (m, 1H), 1.97 – 1.88 (m, 1H), 1.87 – 1.79 (m, 2H), 1.75 – 1.69 (m, 1H), 1.61 – 1.55 (m, 2H), 1.22 – 1.15 (m, 1H), 1.07 (d, J = 6.7 Hz, 3H), 1.04 – 0.99 (m, 6H), 0.93 (t, J = 7.5 Hz, 3H), 0.71 – 0.64 (m, 1H). ^{13}C NMR (126 MHz, Methanol-d_4) δ 173.40 , 173.38 , 172.28 , 172.17 , 172.14 , 172.09 , 171.24 , 170.98 , 170.78 , 169.13 , 156.74 , 137.25 , 136.20 , 128.41 , 128.23 , 128.00 , 127.75 , 126.58 , 126.11 , 61.05 , 60.16 , 59.21 , 57.16 , 54.53 , 50.59 , 47.87 , 47.75 , 46.09 , 45.52 , 45.49 , 40.29 , 37.76 , 36.83 , 34.37 , 31.25 (d, J = 2.9 Hz), 30.46 , 28.79 , 28.30 , 27.41 , 25.52 , 25.45 , 24.82 , 24.15 , 24.09 , 23.57 , 20.29 , 14.08 , 9.50 .

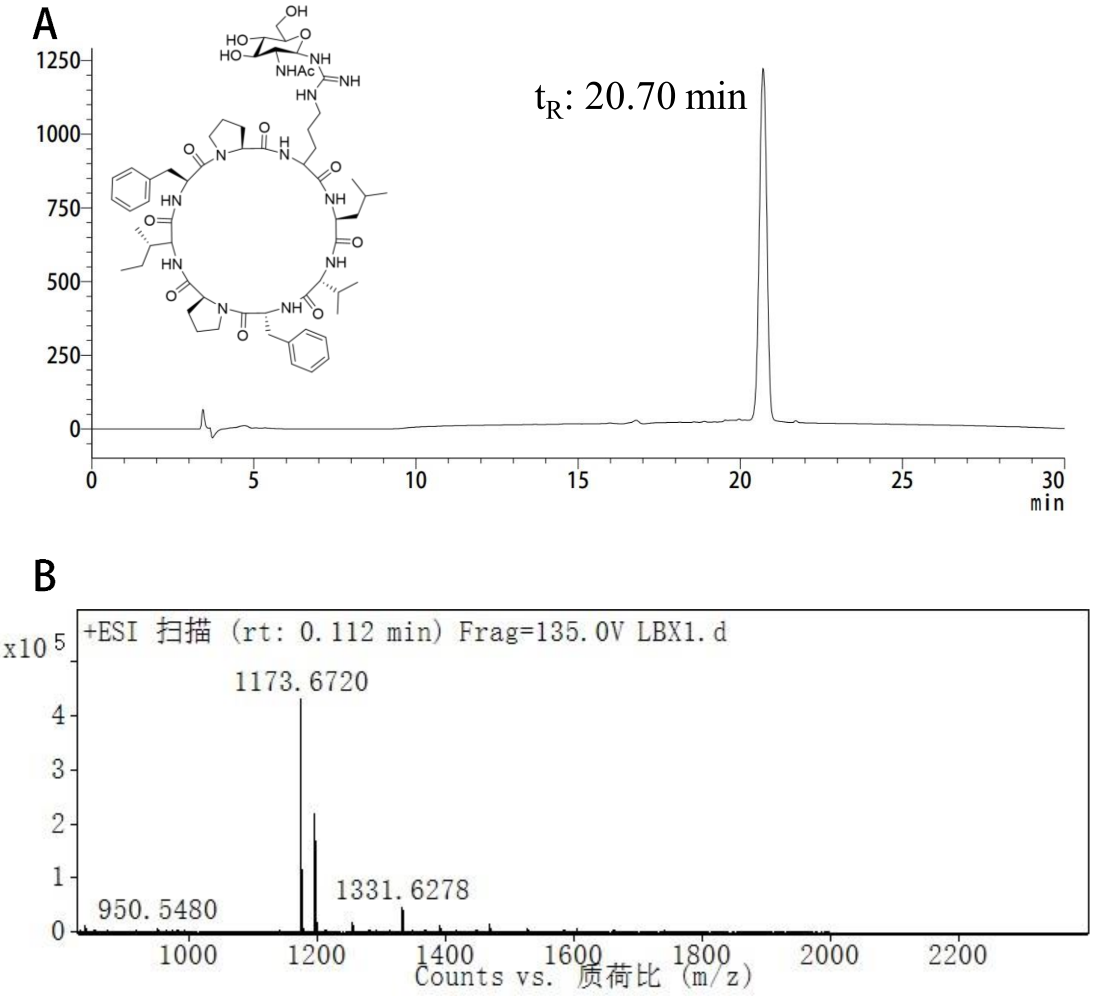

A
tR: 20.70 min
min
B
+ESI 扫描 (rt: 0.112 min) Frag=135.0V LBX1.d
x10 5
1173.6720
1331.6278
950.5480
Counts vs. 质荷比 (m/z)

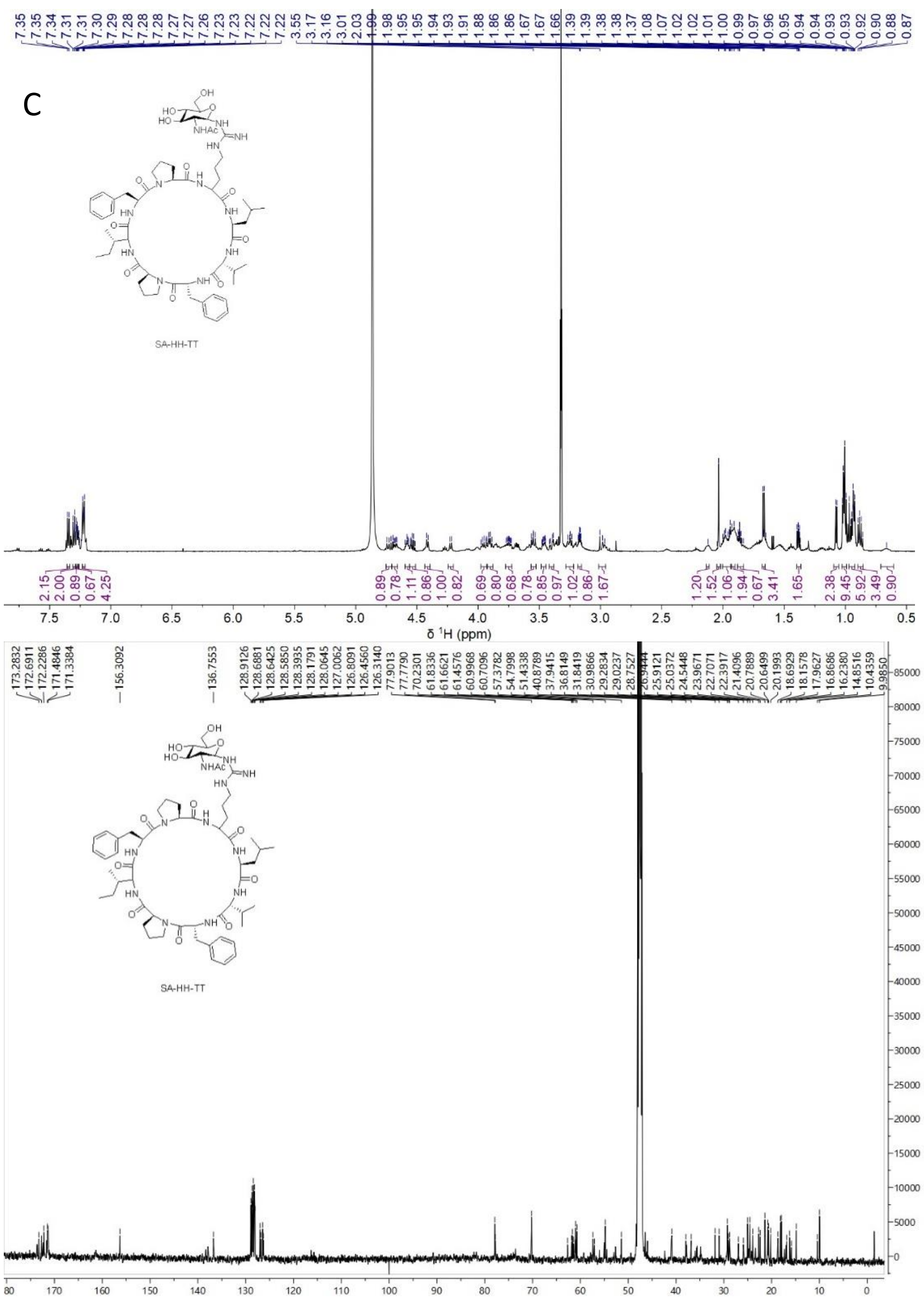

Supplementary Figure 10 A) The HPLC of purified SA-HH-TT; **B)** ESI-MS spectrum of SA-HH-TT ESI-MS m/z calcd. For $C_{59}H_{88}N_{12}O_{13}$ 1172.66; found $[M + H]^+$ = 1173.67; $[M + Na]^+$ =1195.65. **C)** The NMR spectra of SA-HH-TT. ^{1}H NMR (600 MHz, Methanol-*d*$_4$) δ 7.36 – 7.33 (m, 2H), 7.31 – 7.29 (m, 2H), 7.29 – 7.27 (m, 1H), 7.27 – 7.26 (m, 1H), 7.23 – 7.21 (m, 4H), 4.75 – 4.71 (m, 1H), 4.70 – 4.66 (m, 1H), 4.60 – 4.56 (m, 1H), 4.53 (q, *J* = 7.3 Hz, 1H), 4.41 (d, *J* = 7.8 Hz, 1H), 4.22 (d, *J* = 8.9 Hz, 1H), 3.98 – 3.93 (m, 1H), 3.92 – 3.88 (m, 1H), 3.78 – 3.73 (m, 1H), 3.57 – 3.53 (m, 1H), 3.46 (dq, *J* = 8.5, 2.8, 2.3 Hz, 1H), 3.42 – 3.38 (m, 1H), 3.28 – 3.22 (m, 1H), 3.19 – 3.16 (m, 1H), 3.01 – 2.96 (m, 2H), 2.12 (s, 1H), 2.03 (s, 2H), 2.00 – 1.94 (m, 1H), 1.92 (d, *J* = 11.4 Hz, 2H), 1.88 – 1.83 (m, 1H), 1.67 (d, *J* = 7.1 Hz, 3H), 1.40 – 1.36 (m, 2H), 1.07 (d, *J* = 6.5 Hz, 2H), 1.03 – 0.99 (m, 9H), 0.97 – 0.92 (m, 6H), 0.90 – 0.85 (m, 3H), 0.66 (s, 1H). ^{13}C NMR (151 MHz, Methanol-d4) δ 173.28 , 172.69 , 172.23 , 171.34 , 156.31 , 136.76 , 128.91 , 128.71 – 128.57 (m), 128.39 , 128.18 , 128.06 , 127.01 , 126.81 ,

126.46 , 126.31 , 77.90 , 77.78 , 70.23 , 62.67 , 61.83 , 61.66 , 61.46 , 61.00 , 60.71 , 57.38 , 57.06 , 54.80 , 51.43 , 40.88 , 37.94 , 36.81 , 31.84 , 30.99 , 29.28 , 29.02 , 28.75 , 26.94 , 25.91 , 25.04 , 24.54 , 23.97 , 22.71 , 22.39 , 21.41 , 20.79 , 20.65 , 20.20 , 18.69 , 18.16 , 17.96 , 16.87 , 16.24 , 15.87 , 14.85 , 10.44 , 9.99 .

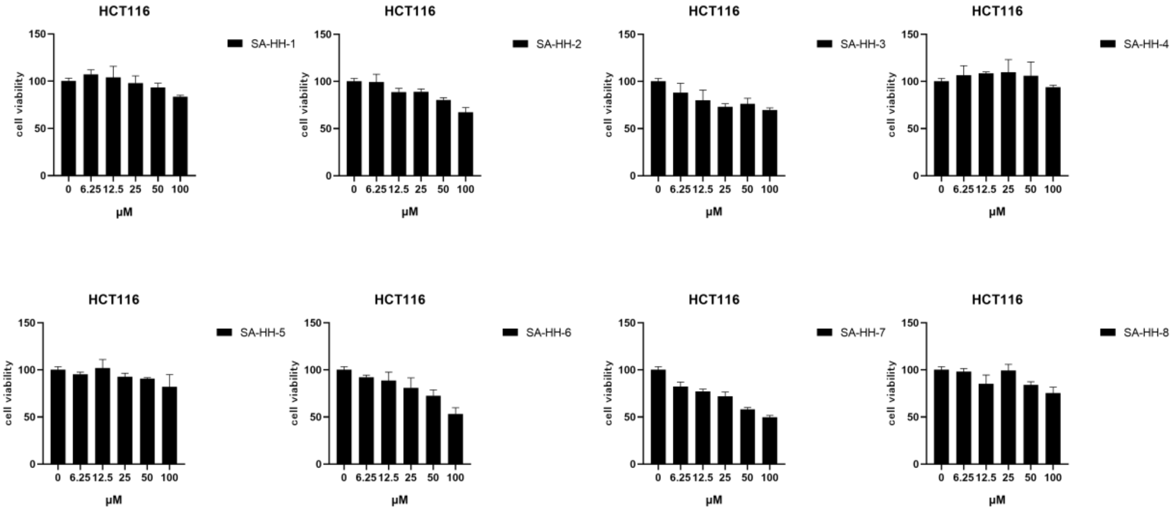

Supplementary Figure 11 Cytotoxicity of arginine scanned derivatives to HCT116

A

Absorbance	Concentration（mg/mL）
0.116	0.04
0.174	0.06
0.226	0.08
0.262	0.1
0.306	0.12
0.35	0.14
0.383	0.16
0.431	0.18
0.466	0.2

B

	Absorbance
**SA-HH-0**	**0.778**
**SA-HH-TT**	**0.059**

Supplementary Figure 12 A) The data of standard curve **B)** The absorbance of the sample diluted 100 times
